# Supplementary material for: The association of cycling with all-cause, cardiovascular and cancer mortality: findings from the population-based EPIC-Norfolk cohort
Source: BMJ Open. 2013 Nov 14;3(11):e003797. doi: 10.1136/bmjopen-2013-003797 (PMC3831097; doi:10.1136/bmjopen-2013-003797)
Supplement: Author's manuscript [file bmjopen-2013-003797.draft_revisions.pdf]

The association of cycling with all-cause, cardiovascular and cancer mortality: findings from the population based EPIC-Norfolk cohort

Journal:	*BMJ Open*
Manuscript ID:	bmjopen-2013-003797
Article Type:	Research
Date Submitted by the Author:	13-Aug-2013

Complete List of Authors:	Sahlqvist, Shannon; Deakin University, Centre for Physical Activity and Nutrition Research (C-PAN); Institute of Public Health, UKCRC Centre for Diet and Activity Research<br>Goodman, Anna; London School of Hygiene and Tropical Medicine, Faculty of Epidemiology and Population Health<br>Simmons, Rebecca; Medical Research Council, Epidemiology Unit<br>Khaw, KayTee; University of Cambridge, Department of Gerontology in Clinical Medicine<br>Cavill, Nick; Cavill Associates,<br>Foster, Charles; University of Oxford, Department of Public Health<br>Luben, Robert; University of Cambridge, Department of Public Health and Primary Care, Institute of Public Health, School of Clinical Medicine<br>Wareham, Nicholas; Medical Research Council, Epidemiology Unit<br>Ogilvie, David; Medical Research Council, Epidemiology Unit

**Primary Subject Heading**:	Epidemiology
Secondary Subject Heading:	Epidemiology, Public health, Sports and exercise medicine
Keywords:	active travel, active commuting, physical activity

SCHOLARONE™ Manuscripts

The association of cycling with all-cause, cardiovascular and cancer mortality: findings from the population based EPIC-Norfolk cohort

Shannon Sahlqvist[1,2,3], Anna Goodman[2,4], Rebecca K. Simmons[1], Kay-Tee Khaw[5], Nick Cavill[6,7], Charlie Foster[7], Robert Luben[8], Nicholas J. Wareham[1,2] & David Ogilvie[1,2]

[1]Medical Research Council Epidemiology Unit, University of Cambridge, UK
[2]UKCRC Centre for Diet and Activity Research (CEDAR), University of Cambridge, UK
[3]Centre for Physical Activity and Nutrition Research (C-PAN), School of Exercise and Nutrition Sciences, Deakin University, Geelong, Australia
[4]Faculty of Epidemiology and Population Health, London School of Hygiene and Tropical Medicine, London, UK
[5]Department of Gerontology in Clinical Medicine, University of Cambridge, UK
[6]Cavill Associates, Stockport, UK
[7]British Heart Foundation Health Promotion Research Group, Nuffield Department of Population Health, University of Oxford, UK
[8]Department of Public Health and Primary Care, Institute of Public Health, University of Cambridge School of Clinical Medicine, Cambridge, UK

Corresponding Author:
Shannon Sahlqvist
Centre for Physical Activity and Nutrition Research (C-PAN)
School of Exercise and Nutrition Sciences
Faculty of Health
Locked Bag 2000
Geelong Victoria 3220 Australia
Email: shannon.sahlqvist@deakin.edu.au
Telephone: 0061 3 9251 7782
Fax: 0061 2 5337 2211

Word Count: 3822
Number of pages: 23
Numbers of tables: 5

For peer review only

ABSTRACT

Objectives: To investigate associations between modest levels of total and domain-specific (commuting, other utility, recreational) cycling and mortality from all causes, cardiovascular disease and cancer.

Design: Population-based cohort study (EPIC-Norfolk).

Setting: Participants were recruited from general practices in the east of England and attended health examinations between 1993 and 1997 and again between 1998 and 2000. At the first health assessment, participants reported their average weekly duration of cycling for all purposes using a simple measure of physical activity. At the second health assessment, participants reported a more detailed breakdown of their weekly cycling behaviour using the EPAQ2 physical activity questionnaire.

Participants: Adults aged 40 – 79 years at the first health assessment.

Primary Outcome Measure: All participants were followed for mortality (all-cause, cardiovascular and cancer) until March 2011.

Results: There were 22,450 participants with complete data at the first health assessment, of whom 4,398 died during follow-up; and 13,346 participants with complete data at the second health assessment, of whom 1,670 died during follow-up. Preliminary analyses using exposure data from the first health assessment showed that cycling for at least 60 min/week in total was associated with a 9% reduced risk of all-cause mortality (adjusted hazard ratio 0.91, 95%CI 0.84, 0.99). Using the more precise measures of cycling available from the second health assessment, although all types of cycling were associated with greater total moderate-to-vigorous physical activity, there was little evidence of an association between overall or domain-specific cycling and mortality.

Conclusions: While this study provides tentative evidence that modest levels of cycling may reduce the risk of mortality, further research is required to confirm how much cycling is sufficient to induce health benefits.

Keywords: active travel, physical activity, commuting

For peer review only

For peer review only - http://bmjopen.bmj.com/site/about/guidelines.xhtml

ARTICLE SUMMARY

Article Focus

Cycling, particularly for transport, is promoted as a way of increasing regular physical activity among adults. Longitudinal studies have demonstrated associations between utility cycling and reduced mortality. However, these associations have been reported only for the highest exposure groups reporting substantial volumes of cycling (i.e. ≥180 min/wk).

We examined associations between mortality and lower volumes of total, and domain-specific, cycling in a population of UK adults.

Key Message

In this population with relatively low levels of cycling, preliminary analyses using a single item to measure total cycling revealed that cycling for as little as 60 min/week in total was associated with a reduced risk of all-cause mortality.

By contrast, in more substantive analyses using a detailed breakdown of cycling behaviour, neither total nor domain-specific cycling were associated with a reduced risk of mortality.

Our results suggest that even modest 'doses' of cycling *may* reduce mortality risk and do not suggest any evidence of an adverse effect, thereby contributing to the growing environmental, social and public health case for promoting cycling in individuals and populations.

Strengths and Limitations of this Study

Strengths of this study include its prospective design, the inclusion of a large heterogeneous population of men and women and the long follow-up. Further, this study used detailed measures of cycling and overall physical activity to examine associations between the various domains of cycling and mortality.

Due to the low average levels of cycling we were not able to examine the specific effects of a higher 'dose' of cycling, and the analyses were underpowered to examine sex differences in the associations between cycling and mortality.

For peer review only - http://bmjopen.bmj.com/site/about/guidelines.xhtml

INTRODUCTION

Promoting cycling as an alternative to motorised transport would result in reduced carbon emissions, traffic congestion and noise pollution while providing people with an opportunity to integrate regular physical activity into their lives.[1,2] As such, there is increasing policy interest in quantifying the health benefits of cycling so that they can be accurately modelled in the economic appraisal of proposed policies and interventions in the transport and health sectors.[3,4] One such tool developed by the World Health Organisation (Health Economic Assessment Tool; HEAT) estimates the economic value of a reduction in mortality as a consequence of population increases in cycling.[5] It does so by assuming a linear dose-response relationship between cycling and mortality and that any increase in cycling is in addition to other physical activity.

HEAT model estimates are dependent on the use of a relative risk estimate from a single study of Danish adults. The study reported a 28% reduction in all-cause mortality in adults who cycled to and from work compared with those who did not, even after controlling for other physical activity.[6] Similarly, an inverse association between transport (utility) cycling more generally and all-cause and cancer mortality has been reported in a cohort of Chinese women.[7] These findings are likely to reflect, in part, the fact that utility cycling translates into greater overall physical activity.[8,9]

While these studies suggest substantial health benefit associated with utility cycling, an examination of the benefits of recreational cycling would also be valuable to enable more informed policy recommendations on which type of cycling to promote.

Furthermore, it is possible that the findings from these studies reflect, at least to some extent, residual confounding from 'other' physical activity. In particular, the Danish study controlled for recreational physical activity using responses to a single item which asked participants to select from one of four options ranging from 'you are almost entirely sedentary or perform light physical activity less than two hours per week' to 'you perform highly vigorous physical activity more than four hours per week or regular exercise or competitive sports several times per week'.[6] The extent to which responses to this item were independent of those regarding commuter cycling was not reported.

In addition, in the two prior studies which reported associations between utility cycling and mortality, the time spent cycling for transport in the exposed groups was substantial, reflecting the relatively high levels of cycling in those countries. For example, in the Danish study, those who commuted by bike spent an average of 180 min/week doing so.[6] In the study of Chinese women, 19% cycled for up to 3.4 Metabolic Equivalents (MET).hr/day while a further 5% cycled for greater than 3.5 MET.hr/day, equivalent to approximately 350 min/week.[7] Few studies have examined associations between cycling and mortality in populations such as that of the UK, which have a low prevalence of utility cycling by international standards. One previous study of adults in the EPIC-Norfolk cohort found no significant association between commuter cycling and either cardiovascular or all-cause mortality.[10] These null findings may partly reflect the cut points used to define cycling categories: the cut point for the highest category was 30 min/week, which may be an insufficient 'dose' to induce health benefits. It is also possible that the relatively short duration of follow-up (seven years) and the small number of deaths in the cohort limited the power of the study to detect effects.

Building on these previous analyses of EPIC-Norfolk cohort data, this paper aims to investigate more comprehensively the mortality benefits of cycling. First, we use a simple pragmatic measure of physical activity to examine associations between total cycling and all-cause, cardiovascular and cancer mortality over 15 years. Second, using a more detailed, disaggregate measure of physical activity which provides more accurate estimates of domain-specific cycling (commuting, all utility, and recreational) for a subset of our sample, we explore whether this association is driven by particular domains of cycling (e.g. utility vs. recreational). Finally, to help explain any associations between domain-specific cycling and mortality, we examine associations between these domains of cycling and total physical activity.

For peer review only - http://bmjopen.bmj.com/site/about/guidelines.xhtml

METHODS

Study design and participants

This study uses data from the EPIC-Norfolk cohort, part of the 10-country collaborative European Prospective Investigation into Cancer and Nutrition study (EPIC). Between 1993 and 1997, 25,633 adults aged 40 – 79 years were recruited from general practices in the county of Norfolk in the east of England and attended a health examination. As part of this examination, participants completed a short physical activity questionnaire which asked about time spent walking and cycling for all purposes and time spent in other exercise.[12] Between January 1998 and October 2000 , 15,519 (61%) of the original cohort attended a second health assessment, completing a more detailed questionnaire on recreational, occupational, utility and household physical activity (EPAQ2).[13]

Data from the first health assessment were used to examine the association between total cycling and mortality, while data from the second were used to examine the association between the domains of cycling and mortality. Full details of the study are reported elsewhere.[11]

Of the participants in the first health assessment, we excluded those with self-reported cardiovascular disease (n=1,102) or cancer (n=1,327) and those with missing data (n=784) leaving 22,450 for analysis.

Similarly, of those who returned for the second health assessment, we excluded those with self-reported cardiovascular disease (n=772) or cancer (n=1,115) and those with missing data (n=286), leaving 13,346 for analysis. All participants were followed up for mortality to 31 March 2011 (mean 15.3 years (SD=3.3) from first health assessment, mean 11.5 years (SD = 2.0) from second health assessment). The Norwich District Health Authority Ethics Committee approved the study design and all participants provided written informed consent.

Health assessments

At both health assessments participants reported their level of education (categorised as no formal qualification; GCSE or equivalent, i.e. exams normally taken at age 16; 'A' level or equivalent, i.e. exams normally taken at age 18; university degree or equivalent), paid employment status (yes, no), social class (categorised as professional, managerial/technical job, skilled/partially skilled labour, unskilled labour), smoking status (current, former, never), anti-hypertensive medication (yes, no), medication for dyslipidaemia (yes, no) and family history of cancer and cardiovascular disease (yes, no). History of myocardial infarction, stroke, and cancer were also reported. Total energy intake (kJ/day) and alcohol consumption (units/week) were derived from a validated 130-item semi-quantitative food-frequency questionnaire.[14]

Measurement of physical activity at first health assessment

Physical activity was assessed by asking participants to report, separately for winter and summer, the weekly time (in hours) spent walking and cycling (separately) to work and during leisure, and in other exercise.[12] Total cycling was calculated as the average weekly time spent in winter and summer (min/week). See Appendix, part 1.

Measurement of physical activity at second health assessment

Physical activity, including cycling, was assessed with the validated and reliable EPAQ2 questionnaire, which asks participants to recall their physical activity behaviour across the domains of household, work, recreation and commuting, over the past year.[13] Energy expenditure [MET.hr/week] was calculated using the physical activity compendium.[15] Following standard EPAQ2 data reduction rules we calculated four specific cycling measures explained in detail in Table 1. In addition, total moderate-to-vigorous physical activity was calculated as the sum of all moderate and vigorous physical activity across all domains (home, work, recreation and commuting; MET.hr/week) and recreational physical activity was computed as the sum of all moderate and vigorous activity done during recreation specifically (MET.hr/week). A copy of the questionnaire can be found at: http://www.srl.cam.ac.uk/epic/questionnaires/epaq2/epaq2.pdf.

For peer review only - http://bmjopen.bmj.com/site/about/guidelines.xhtml

[Insert Table 1 about here]

Cycling exposure

Given the highly skewed nature of the cycling data and to allow for comparisons with previous studies, we created three categories of cycling exposure: 0 min/week, 1 – 59 min/week and ≥ 60 min/week. These represent levels of cycling which we believe are realistic to achieve in countries such as the UK, which currently have low levels of utility cycling. For our measures of utility cycling from the second health assessment these categories are equivalent to: 0 miles/week; 0.01– 9.99 miles/week and ≥ 10 miles/week.

Mortality outcomes

All EPIC-Norfolk participants were flagged for death certification with the UK Office of National Statistics (ONS). Trained nosologists coded death certificates according to the ICD-9 or ICD-10. Cardiovascular death was defined as ICD 410–448 (ICD 9) or ICD I10–I79 (ICD 10) as underlying cause of death, which comprise coronary heart disease (410–414 (ICD 9) or I20–I25 (ICD 10)), stroke (430–438 (ICD 9) or I60–I69 (ICD 10)), cardiac failure (428 (ICD 9) or I50 (ICD 10)) and other vascular causes. Cancer death was defined as ICD 140-208 (ICD9) or ICD C00 – C97 (ICD10) as the underlying cause.

Statistical analysis

We used exposure data from the first health assessment to examine associations between total cycling and all-cause, cardiovascular and cancer mortality. Exposure data from the second health assessment were used to explore associations between total, and domain specific cycling and mortality and overall physical activity. First, using data collected from the first health assessment, we examined preliminary associations between total cycling and all-cause, cardiovascular and cancer morality by fitting Cox proportional hazard regression models to estimate hazard ratios and 95% confidence intervals. We first adjusted for sex, age, education level and social class (Model A) and then further adjusted for smoking status, family history of cardiovascular disease or cancer, as well as time spent walking and in other exercise. As sensitivity analyses, we ran a further two models. In the first we adjusted for weekly alcohol consumption and calorie intake; 4% (n=912) of participants had missing data for these variables. In the second, we further adjusted for medication (hypertension and dyslipidaemia) and type 2 diabetes as we thought it possible they could be mediating variables on the causal pathway between cycling and mortality. Results of these sub-group analyses did not differ substantially from those of Model B and are not presented. Models were also run after excluding participants who died within two years of follow-up (n=181) to minimise the potential effect of reverse causality. This made no substantive differences to the findings (data not presented).

We then examined the associations between the domains of cycling and mortality, again by fitting Cox proportional hazard regression models. Equivalent models to those described above were run except that Model B also controlled for all other physical activity energy expenditure (calculated as the sum of all energy expenditure in all domains of physical activity minus that of the respective cycling behaviour). To account for the potentially conservative estimates of commuting cycling undertaken when cycling was selected alongside other modes (see Table 1), by way of sensitivity analysis we applied an alternative assumption that commuter cycling was done for 30% (rather than 10%) of these journeys. Findings remained largely unchanged when using these new estimates. Again, our results were substantively unchanged after adjusting for weekly alcohol consumption and calorie intake, after further adjustment for medication and type 2 diabetes, or after excluding the 102 participants who died within two years of follow-up (data not presented).

For all models the proportional hazard assumption was verified using Schoenfeld residuals and Kaplan-Meier plots for all three outcomes. For all models, we also present p-values for linear trend, calculated by entering the domains of cycling as continuous rather than categorical variables.

For peer review only - http://bmjopen.bmj.com/site/about/guidelines.xhtml

To examine whether any observed associations between cycling and mortality could be explained by differences in overall levels of physical activity, we examined associations between the domains of cycling and physical activity (total leisure-time, total moderate-to-vigorous across all domains, and total light, moderate and vigorous across all domains) by fitting linear regression models with time spent cycling (total and sub domains) as the exposure variables and time spent in (a) recreational and (b) total moderate-to-vigorous physical activity (MET.hr/week) as the outcome variables controlling for sex, age, social class and highest level of education. All analyses were conducted using STATA, version 12.0 (Stata Corp., TX, USA).

RESULTS

Participant characteristics

At the first health assessment, participants had a mean age of 58 years (SD=19) and just over half were women (55%). 24% of participants reported cycling for a mean of 165 min/week (SD=246). Socio-demographic characteristics of the cohort by cycling status (yes, no) are described in Table 2 (for further details of the baseline characteristics of the sample see Appendix, part 2, Table A1). Respondents who reported any cycling were, on average, younger and more likely to be men. Respondents with no formal qualification were also more likely to cycle compared with respondents with GCSE-level qualifications, while those in skilled or unskilled labour were more likely to cycle than professionals.

[Insert Table 2 about here]

By the second health assessment participants had a mean age of 62 (SD 9) years; just over half were women (57%). 30% (n=4030) reported any cycling. Of those who cycled, 62% (n=2808) reported cycling for recreation and 72% (n=3269) reported cycling for utility purposes with 26% (n=862) of these reporting commuting cycling. The average cyclist spent 83 min/week cycling. Those who commuted by bicycle spent an average of 61 min/week doing so, while those who cycled for recreation spent an average of 58 min/week doing so. Again, men and those who were younger were more likely to cycle. In addition to the sociodemographic associations observed in data from the first health assessment, respondents working in a managerial/technical position were less likely to cycle than professionals. Employment status also showed a strong association with cycling, probably reflecting the fact that commuting was included in the measure of cycling (see Appendix, part 2, Table A2).

Total cycling (first health assessment) and mortality

4,398 (20%) participants died during 3,425,498 person-years of follow-up (Table 3). There were 1,379 (6.1%) cardiovascular deaths and 1,639 (7.3%) cancer deaths (see Appendix, Part 2, Table A3). Risk of death was associated with being men, older, and having a lower level of education and social class.

Cycling for at least 60 min/week was associated with a 9% reduction in all-cause mortality after controlling for potential confounders (HR 0.91, 95% CI 0.84, 0.99; Table 3). This seemed to be driven by the protective association between cycling and cardiovascular mortality (HR 0.85, 95% CI 0.72, 0.99). There were no significant associations between cycling and cancer mortality.

[Insert Table 3 about here]

Domains of cycling (second health assessment) and mortality

1,670 (12.5%) individuals died during 149,072 person-years of follow-up. There were 485 (3.6%) cardiovascular deaths and 700 (5.2%) cancer deaths. Again mortality rates were higher among men and older participants (data not shown). There were no significant associations between commuting cycling and all-cause, cardiovascular or cancer mortality in either the minimally adjusted (A) or the additionally adjusted (B) models (Table 4). For both all-cause and cancer mortality, however, there was suggestion of a dose-response relationship between distance cycled and risk of death whereby the lowest hazard ratios were observed for the highest levels of commuting

For peer review only - http://bmjopen.bmj.com/site/about/guidelines.xhtml

cycling, albeit not reaching statistical significance. There was no association between all utility cycling and all-cause, cardiovascular or cancer mortality in either the minimally adjusted (A) or the additionally adjusted (B) models. In minimally adjusted models, recreational cycling for less than 60 min/week was associated with a 19% reduced risk (95% CI 0.66, 0.99). Further adjusted attenuated the effect. There were no significant associations between total cycling and mortality.

[Insert Table 4 about here]

Association between domains of cycling and total physical activity (second health assessment)

Total and domain-specific cycling was associated with greater levels of physical activity in an approximately dose-response relationship (Table 5). All utility, recreation and total, but not commuting, cycling were associated with greater recreational physical activity. Importantly, however, commuting cycling was not inversely associated with recreational physical activity, suggesting that adults were not cycling to and from work to compensate for a lack of recreational physical activity. The association between cycling and recreational physical activity was strongest for recreational cycling; those who spent 1 to 59 min/week cycling for pleasure participated in an additional 3 MET.hr/day of recreational physical activity (equivalent to approximately 36 min/day of moderate intensity physical activity).

All domains of cycling showed significant dose-response relationships with total moderate-to-vigorous physical activity, although the association was strongest for commuting cycling. Those who cycled for ≥60 min/week spent an additional 7.9 MET.hr/day in moderate-to-vigorous physical activity, (equivalent to 94.8 min/day of moderate intensity physical activity) compared with those who did not.

[Insert Table 5 about here]

DISCUSSION

We used data from a large population-based cohort to examine the associations between total and domain-specific cycling and mortality. Across all domains, cyclists were more likely to be younger and men, a finding that is consistent with previous studies conducted in countries that have low rates of utility cycling[16-18] but different from the pattern in a number of other European countries where men and women, and the young and old, are equally likely to cycle.[19]

Using exposure data from the first health assessment, cycling for at least 60 min/week in total was associated with a 9% reduction in risk of all-cause, and a 15% reduction in risk of cardiovascular, mortality but was not associated with cancer mortality. In the absence of any directly comparable data on total cycling from other studies, these findings provide tentative evidence that modest ‘doses’ of cycling may be associated with a reduction in mortality risk. They are also broadly consistent with the findings of the Danish study in which a reduction in mortality risk (28%) was associated with an average quantity of cycling that was three times higher (180min/week).[6]

That being said, when using more precise measures of cycling we found no significant associations between total or domain-specific cycling and mortality. This was despite the fact that all domains of cycling, and commuter cycling in particular, were associated with higher levels of overall physical activity. On the one hand, these differences could reflect a lack of power in analyses of the second health assessment data, which included fewer participants and had a shorter follow-up period. On the other hand, the more precise measures of physical activity may have not only enabled more accurate categorisation of cycling exposure, but also reduced measurement error regarding the confounding effect of ‘other’ physical activity.

Despite five additional years of follow-up and the examination of a higher ‘dose’ of cycling, our null findings relating to the mortality benefits of commuting and utility cycling in particular mirror those previously reported in this cohort[10] and are consistent with those of previous studies of low-cycling populations in Northern Ireland and

For peer review only - http://bmjopen.bmj.com/site/about/guidelines.xhtml

France, which found no evidence of a reduced risk of fatal or nonfatal myocardial infarction in men who reported any walking or cycling to work compared with those who did not.[20]

They are however in contrast to the findings of the studies of Danish[6] and Chinese[7] adults and of a meta-analysis, which pooled evidence from eight studies (from five independent populations) and found that active commuting (walking and cycling) was associated with an overall 11% reduction in the risk of cardiovascular outcomes.[21] Importantly, the levels of commuting cycling reported by participants in these previous studies were substantial and in the meta-analysis, evidence of protective effects was generally limited to higher levels of active commuting.[21] The high 'doses' of utility cycling reported in previous studies are likely to be achieved when cycling journeys are taken frequently and consistently (e.g. twice daily, five days per week). It is possible that frequent short bursts of physical activity of this kind are beneficial to health in their own right, rather than simply by contributing to greater levels of total physical activity as we have shown. In support of this hypothesis, studies have demonstrated that accumulated short bouts of exercise over the day result in longer post-exercise reductions in blood pressure[22] and lower plasma triglycerides[23] than one continuous session of exercise. There is also some evidence that the intensity of cycling is important. A study of Danish adults found a significant inverse association between cycling intensity and all-cause and coronary heart disease mortality,[24] and it may be that participants in our study were not cycling at an intensity sufficient to result in health benefit. It is also possible that the differences reflect the fact that our cohort was older than the Danish and Chinese cohorts.

To further elucidate the health benefits of cycling and refine the use of tools such as HEAT that may be used to inform policy in this area, future research should aim to estimate the association between cycling and mortality independent of other physical activity, measured with as little error as possible; to extend such analyses to include morbidity endpoints such as incident cardiovascular disease and diabetes; and to clarify how much cycling is sufficient to induce health benefits by quantifying the mean quantity (and preferably intensity) of cycling in each exposure category studied and describing the shape of the dose-response relationship. In the meantime, our results suggest that even modest 'doses' of cycling *may* reduce mortality risk and do not suggest any evidence of an adverse effect, thereby contributing to the growing environmental, social and public health case for promoting cycling in individuals and populations.

Strengths and limitations

This is the first study to examine independent associations between total and domain-specific cycling and mortality. Other strengths of the study include its prospective design, the inclusion of a large heterogeneous population of men and women and its long follow-up. We adjusted our analyses of the second health assessment data for all types of physical activity as well as a range of potential demographic and behavioural confounders which strengthens the inferences made. Excluding participants with existing chronic disease and those who died within two years of follow-up enabled us to control for reverse causality. Given the population-based recruitment from a large geographical area, we believe that our findings are generalizable to middle and older aged adults. There are, however, a number of limitations. Cycling and total physical activity were assessed by self-report. The cycling exposure variables, in particular utility cycling, were derived from relatively crude measures and assumptions had to be made about frequency of cycling, distance travelled and average speed. Due to the low average levels of cycling, we were not able to examine the specific effects of a high 'dose' of cycling and the analyses were underpowered to examine sex differences in the associations which have been previously documented.[21]

Conclusions

Building on previous research that demonstrated inverse associations between high doses of utility cycling and mortality, we used data from a large, population-based cohort to examine associations between more modest levels of cycling and mortality. Our preliminary findings add tentative support to the hypothesis that lower levels of cycling are also associated with a reduced risk of mortality compared with not cycling at all. However, this finding was not replicated in subsequent analyses using more detailed measures of exposure, albeit in fewer

For peer review only - http://bmjopen.bmj.com/site/about/guidelines.xhtml

participants who were followed up for a shorter period. Nevertheless, the positive association between cycling in general, and commuting cycling in particular, and overall physical activity suggests that encouraging people to cycle is likely to have considerable health benefits.

For peer review only

For peer review only - http://bmjopen.bmj.com/site/about/guidelines.xhtml

Acknowledgements: We thank Dr Søren Brage and Dr Marcel Den Hoed for their assistance in cleaning the EPAQ2 data.

Contributors: DO and CF conceived the idea of the analysis and SS and DO designed the analysis with advice from the other authors. SS analysed the data and wrote the initial draft of the manuscript with DO, AG and RKS. KTK, NW and RL contributed to the design of the EPIC-Norfolk study protocol. All authors contributed to the critical review of the design of the analysis and the critical revision of the manuscript and approved the final version.

Funding: DO is supported by the Medical Research Council (Unit Programme number MC_UP_1001/1) and by the Centre for Diet and Activity Research (CEDAR), a UKCRC Public Health Research Centre of Excellence which also hosted SS and AG. Funding from the British Heart Foundation, Economic and Social Research Council, Medical Research Council, the National Institute for Health Research, and the Welcome Trust, under the auspices of the UK Clinical Research Collaboration, is gratefully acknowledged. AG is supported by a National Institute of Health Research (NIHR) post-doctoral fellowship. The views and opinions expressed in this article are those of the authors and do not necessarily reflect those of the NIHR, the Department of Health or other study funders. The EPIC-Norfolk study is funded by the Cancer Research Campaign; the Medical Research Council, the Stroke Association, the British Heart Foundation, the Department of Health, the Europe Against Cancer Programme Commission of the European Union and Ministry of Agriculture, Fisheries and Food. The authors thank the EPIC-Norfolk staff and participants for their valuable contributions.

Competing Interests: None

Ethics Approval: The Norwich District Health Authority Ethics Committee approved the study design and all participants provided written informed consent.

For peer review only

REFERENCES

1. Woodcock J, Banister D, Edwards P, Prentice AM, Roberts I. Energy and Health 3. *Lancet* 2007;370(9592):1078-88.
2. Shepard R. Is active commuting the answer to population health? *Sports Med* 2008;38(9):751-58.
3. Jarrett J, Woodcock J, Griffiths UK, Chalabi Z, Edwards P, Roberts I, et al. Effect of increasing active travel in urban England and Wales on costs to the National Health Service. *Lancet* 2012;379:2198-205.
4. Woodcock J, Givoni M, Morgan AS. Health Impact Modelling of Active Travel Visions for England and Wales Using an Integrated Transport and Health Impact Modelling Tool (ITHIM). *Plos One* 2013;8(1):e51462.
5. Kahlmeier S, Cavill N, Dinsdale H, Rutter H, Götschi T, Foster C, et al. Health economic assessment tools (HEAT) for walking and for cycling: World Health Organization, 2011.
6. Andersen L, Schnohr P, Schroll M, Hein A. All-cause mortality associated with physical activity during leisure time, work, sports, and cycling to work. *Arch Intern Med* 2000;160:1621-28.
7. Matthews CE, Jurj AL, Shu X, Li H-L, Yang G, Li Q, et al. Influence of exercise, walking, cycling and overall nonexercise physical activity on mortality in Chinese women. *Am J Epidemiol* 2007;165(12):1343-50.
8. Sahlqvist S, Song Y, Ogilvie D. Is active travel associated with greater physical activity? The contribution of commuting and non-commuting active travel to total physical activity in adults. *Prev Med* 2012;55(3):206-11.
9. Sahlqvist S, Goodman A, Cooper A, Ogilvie D, on behalf of the iConnect constortium. Change in active travel and changes in recreational and total physical activity in adults: longitudinal findings from the iConnect study. *Int J Behav Nutr Phys Act* 2013;10(1):28.
10. Besson H, Ekelund U, Brage S, Luben R, Bingham S, Khaw K-T, et al. Relationship between subdomains of total physical activity and mortality. *Med Sci Sport Exer* 2008;40(11):1909-15.
11. Day N, Oakes S, Luben R, Khaw K-T, Bingham S, Welch A, et al. EPIC Norfolk: Study design and characteristics of the cohort. *Br J Cancer* 1999;80(Supplement 1):95-103.
12. Khaw K-T, Jakes R, Bingham S, Welch A, Luben R, Day N, et al. Work and leisure time physical activity assessed using a simple, pragmatic, validated questionnaire and incident cardiovascular disease and all-cause mortality in men and women: The European Prospective Investigation into Cancer in Norfolk prospective population study. *Int J Epidemiol* 2006;35(4):1034-43.
13. Wareham N, Jakes R, Rennie K, Mitchell J, Hennings S, Day N. Validity and repeatability of the EPIC-Norfolk Physical Activity Questionnaire. *Int J Epidemiol* 2002;31:168-74.
14. Bingham S, Gill C, Welch A, Cassidy A, Runswick S, al e. Validation of dieatary assessment methods in the UK arm of EPIC using weighted records, and 24-hour urinary nitrogen and potassium and serum vitamin C and carotenoids as biomarkers. *Int J Epidemiol* 1997;26:S137-S51. .
15. Ainsworth BE, Haskell WL, Leon A, Jacobs DJ, Montoye H, Sallis J, et al. Compendium of physical activities: classification of energy costs of human physical activities. *Med Sci Sport Exer* 1993;25(10):1192.
16. Sahlqvist S, Heesch K. Characteristics of utility cyclists in Queensland, Australia: An examination of the association between individual, social and environmental factors and utility cycling. *J Phys Act Health* 2012;9:818-28.
17. Winters M, Friesen MC, Koehoorn M, Teschke K. Utilitarian bicycling: A multilevel analysis of climate and personal influences. *Am J Prev Med* 2007;32(1):52-58.
18. Foster C, Panter J, Wareham N. Assessing the impact of road traffic on cycling for leisure and cycling to work. *Int J Behav Nutr Phys Act* 2011;8(1):61.
19. Pucher J, Buehler R. Making cycling irresistible: Lessons from The Netherlands, Denmark and Germany. *Transport Reviews* 2008;28(4):495-528.
20. Wagner A, Simon C, Evans A, Ferrières J, Montaye M, Ducimetière P, et al. Physical activity and coronary event incidence in Northern Ireland and France. *Circulation* 2002;105(19):2247-52.
21. Hamer M, Chida Y. Active commuting and cardiovascular risk: a meta-analytic review. *Prev Med* 2008;46:9-13.
22. Park S, Rink LD, Wallace JP. Accumulation of physical activity leads to a greater blood pressure reduction than a single continuous session, in prehypertension. *J Hyper* 2006;24:1761-70.
23. Altena TS, Michaelson JL, Ball SD, Thomas TR. Single sessions of intermittent and continuous exercise and postprandial lipemia. *Med Sci Sport Exer* 2004;36(8):1364-71.
24. Schnohr P, Marott JL, Jensen JS, Jensen GB. Intensity versus duration of cycling, impact on all-cause and coronary heart disease mortality: the Copenhagen City Heart Study. *Euro J Prev Cardiol* 2012;19(1):73-80.

For peer review only - http://bmjopen.bmj.com/site/about/guidelines.xhtml

25. Goodman A, Brand C, Ogilvie D. Associations of health, physical activity and weight status with motorised travel and transport carbon dioxide emissions: a cross-sectional, observational study. *Environ Health* 2012;11(1):52.

For peer review only

For peer review only - http://bmjopen.bmj.com/site/about/guidelines.xhtml

431 TABLES

432 **Table 1: Cycling exposure measures calculated from the EPAQ2 questionnaire administered at the**
433 **second health assessment**

Exposure	Calculation
Commuter cycling	Respondents were asked how frequently they normally travelled to work by car, public transport, bike or on foot (response options were 'always', 'usually', 'occasionally' or 'never/rarely'). Responses were converted to fractions (always = 1, usually = 0.75, occasionally = 0.25, never / rarely = 0). Participants reported the distance between home and work and the average number of times per week they made this journey (multiplied by two to account for the return journey). When cycling was the only mode selected, total weekly distance cycled was calculated by multiplying the distance from home to work by the number of journeys made. When cycling was selected alongside other modes, the distance cycled was weighted according to the frequency of cycling relative to the frequency of the other modes reported. For example, if a respondent selected 'always' for both cycling and driving it was assumed that cycling accounted for 10%, and driving for 90% of the distance travelled. Total number of journeys was then multiplied by the weighted distance travelled (miles/week).
Non-commuting utility cycling	Respondents were asked to recall the average number of journeys they made by bicycle to get about apart from going to work for each of the following distances: 'less than 0.5 miles', '0.5 miles to 1.5 miles', '1.5 to 2.5 miles', '2.5 to 3.5 miles', '3.5 to 5.5 miles', and 'more than 5.5 miles'. Total weekly distance travelled was computed by multiplying the reported number of trips by the midpoint value of each distance category (assumed to be 0.25 for <0.5 miles and 6 for >5.5 miles). These values were then summed to provide a measure of distance travelled (miles/week).
All utility cycling	Distance travelled for non-commuting utility cycling was added to distance travelled for commuting cycling to derive a measure of total utility cycling (miles/week).
Recreational cycling	Respondents reported the average time spent 'cycling for pleasure' per session and the frequency of such sessions: 'none', 'less than once a month', 'once a month', 'two to three times a month', 'once a week', 'two to three times a week', 'four to five times a week', or 'everyday'. Average weekly cycling duration was computed by converting the frequency into a weekly numerical value (e.g. 0.5/52 for 'less than once a month' and (2.5*12)/52 for '2 to 3 times per month'). Time spent cycling (min/week) was computed by multiplying the average session duration by the average weekly frequency.
Total cycling	To enable a measure of total cycling to be derived and to allow for comparisons with previous studies the distance travelled for utility cycling was converted into an estimated duration. Based on self-report data from a recent study of UK adults, we assumed an average cycling speed of 10 miles/hour.[25] A measure of total time spent cycling (min/week) was derived by summing time spent in commuting, other utility and recreational cycling.

434
435

For peer review only - http://bmjopen.bmj.com/site/about/guidelines.xhtml

436 **Table 2: Descriptive characteristics of sample [N (%)] at first heath assessment (n=22,450) by cycling**
437 **(yes, no)**

Characteristic	0 min/week: N (column %)	≥ 1 min/week: N (column %)	OR for any cycling (95%CI)[a]
Sex			
Man	3880 (66.9)	1920 (72.0)	1.0
Woman	5436 (33.1)	2110 (28.0)	0.92 (0.86, 0.98)
Age (yrs)			
40 – 55	2096 (58.1)	1498 (41.7)	1.0
50 – 65	3105 (67.4)	1473 (32.2)	0.72 (0.67, 0.78)
≥65	4115 (79.4)	1059 (20.5)	0.44 (0.40, 0.49)
Education Level			
Degree or equivalent	1280 (65.6)	670 (34.4)	1.0
'A' Level or equivalent	3867 (69.2)	1719 (30.8)	0.91 (0.81, 1.02)
GCSE or equivalent	1054 (71.0)	431 (29.0)	1.02 (0.95, 1.11)
No formal qualification	3115 (72.09)	1210 (28.0)	1.29 (1.15, 1.44)
Social Class			
Professional	665 (66.4)	336 (33.6)	1.0
Managerial / technical	5269 (71.8)	2071 (28.2)	0.90 (0.79, 1.02)
Skilled / partially skilled labour	3078 (67.7)	1469 (32.3)	1.15 (1.00, 1.31)
Unskilled labour	304 (66.4)	154 (33.6)	1.36 (1.08, 1.64)
Paid Employment			
No	8578 (81.0)	2080 (19.0)	1.0
Yes	8365 (72.5)	2127 (27.2)	1.05 (0.97, 1.14)

438 [a]Adjusted for all other variables in the table
439 OR = odds ratio, CI = 95% confidence interval
440

For peer review only

Table 3: Prospective associations over 15 years between total cycling and mortality (all-cause, cardiovascular and cancer) in 22,450 participants

Total cycling	FU yrs Mean (SD)	All-cause mortality Events N (%)	All-cause mortality Hazard Ratio (95%CI) Model A[a]	Model B[b]	Cardiovascular mortality Events N (%)	Cardiovascular mortality Hazard Ratio (95%CI) Model A[a]	Model B[b]	Cancer mortality Events N (%)	Cancer mortality Hazard Ratio (95%CI) Model A[a]	Model B[b]
0 min/week	15.2 (3.4)	3,686 (21.3)	1	1	1179 (6.8)	1	1	1352 (7.8)	1	1
1 – 59 min/week	15.7 (2.6)	100 (10.8)	0.86 (0.71, 1.07)	0.96 (0.78, 1.17)	25 (2.7)	0.73 (0.49, 1.08)	0.83 (0.56, 1.24)	44 (4.7)	0.91 (0.68, 1.24)	0.99 (0.73, 1.34)
≥60 min/week	15.7 (3.0)	612 (14.3)	0.86 (0.79, 0.94)**	0.91 (0.84, 0.99)*	179 (4.2)	0.81 (0.69, 0.95)*	0.87 (0.74, 1.02)*	252 (5.9)	0.89 (0.77, 1.01)	0.93 (0.81, 1.06)
p for linear trend			0.02	0.06		0.04	0.09		0.20	0.23

*p <.05, **p < .01, ***p<.001; 95% CI: 95% confidence interval; FU: follow-up

[a]Adjusted for age, sex, education level and social class

[b]Further adjusted for smoking status, family history of cancer or cardiovascular disease, and other physical activity (walking and other exercise)

For peer review only

For peer review only - http://bmjopen.bmj.com/site/about/guidelines.xhtml

Table 4: Prospective association over 11.5 years between cycling (total and domain specific) and mortality (all-cause, cardiovascular and cancer) in 13,346 participants

	FU yrs Mean (SD)	All-cause mortality			Cardiovascular mortality			Cancer mortality		
		Events N (%)	Hazard Ratio (95%CI)		Events N (%)	Hazard Ratio (95%CI)		Events N (%)	Hazard Ratio (95%CI)	
			Model A[a]	Model B[b]		Model A[a]	Model B[b]		Model A[a]	Model B[b]
Commuting										
0 miles/week (0 min/week)	11.1 (2.0)	1630 (13.1)	1	1	474 (3.8)	1	1	679 (5.4)	1	1
0.01-9.99 miles/week (1-59 min/week)	11.5 (1.6)	29 (4.9)	0.87 (0.60, 1.26)	0.96 (0.66, 1.39)	9 (1.5)	1.09 (0.56, 2.13)	1.23 (0.63, 2.40)	16 (2.7)	0.90 (0.55, 1.49)	0.95 (0.57, 1.57)
≥10 miles/week (≥60 min/week)	11.5 (1.4)	11 (4.0)	0.80 (0.44, 1.46)	0.91 (0.50, 1.65)	2 (0.7)	0.61 (0.15, 2.47)	0.71 (0.18, 2.90)	5 (1.8)	0.66 (0.27, 1.59)	0.68 (0.28, 1.66)
p for linear trend			0.42	0.74		1.00	0.72		0.28	0.34
All Utility										
0 miles/week 0 min/week)	11.1 (2.0)	1383 (13.2)	1	1	392 (3.8)	1	1	580 (5.5)	1	1
0.01-9.99 miles/week (1-59 min/week)	11.6 (1.8)	233 (10.4)	0.90 (0.78, 1.04)	0.95 (0.83, 1.09)	75 (3.4)	1.04 (0.81, 1.34)	1.10 (0.85, 1.41)	97 (4.3)	0.85 (0.69, 1.06)	0.90 (0.72, 1.11)
≥10 miles/week (≥60 min/week)	11.4 (1.8)	54 (8.4)	1.01 (0.77, 1.33)	1.10 (0.83, 1.44)	18 (2.8)	1.30 (0.81, 2.10)	1.44 (0.89, 2.31)	23 (3.6)	0.89 (0.58, 1.35)	0.92 (0.61, 1.41)
p for linear trend			0.71	0.33		0.81	0.52		0.94	0.89
Recreational										
0 min/week	11.1 (2.1)	1483 (13.7)	1	1	438 (4.0)	1	1	608 (5.6)	1	1
1 – 59 min/week	11.4 (1.5)	104 (5.9)	0.81 (0.66, 0.99)*	0.87 (0.69, 1.04)	25 (1.4)	0.72 (0.48, 1.09)	0.75 (0.50, 1.13)	56 (3.2)	0.90 (0.68, 1.19)	0.95 (0.71, 1.25)
≥60 min/week	11.3 (1.8)	83 (11.1)	1.13 (0.90, 1.41)	1.25 (0.99, 1.55)	22 (3.0)	1.07 (0.69, 1.65)	1.19 (0.77, 1.84)	36 (4.8)	1.06 (0.75, 1.49)	1.12 (0.80, 1.58)
p for linear trend			0.12	0.05		0.48	0.32		0.46	0.35
Total cycling										
0 min/week	11.1 (2.1)	1308 (14.0)	1	1	379 (4.1)	1	1	540 (5.8)	1	1
1 – 59 min/week	11.5 (1.8)	236 (8.9)	0.87 (0.76, 1.00)*	0.90 (0.79, 1.04)	72 (2.7)	0.95 (0.74, 1.23)	0.99 (0.76, 1.23)	105 (4.0)	0.86 (0.70, 1.06)	0.90 (0.73, 1.11)
≥60 min/week	11.4 (1.7)	126 (9.2)	1.00 (0.83, 1.21)	1.11 (0.91, 1.32)	34 (2.5)	1.00 (0.70, 1.43)	1.10 (0.77, 1.57)	55 (4.0)	0.92 (0.69, 1.22)	0.98 (0.74, 1.30)
p for linear trend			0.08	0.26		0.36	0.18		0.81	0.51

*p <.05, **p < .01, ***p<.001; 95% CI: 95% confidence interval; FU: follow-up

[a]Adjusted for age, sex, education level and social class

[b]Further adjusted for smoking status, family history of cancer or cardiovascular disease, and all other physical activity

For peer review only

For peer review only - http://bmjopen.bmj.com/site/about/guidelines.xhtml

Table 5: Associations between time spent cycling (total and sub domains; min/week) and physical activity (MET.hr/week) in 13,346 participants

		Leisure Time PA[a] (MET.hr/week)		Total MVPA[b] (MET.hr/week)		Total PA[c] (MET.hr/week)	
	N	Mean (SD)	Regression coefficient (95% CI)[d]	Mean (SD)	Regression coefficient (95% CI)[d]	Mean (SD)	Regression coefficient (95% CI)[d]
Commuter cycling							
0 miles/week (0 min/week)	12484	39.4 (37.7)	0	61.9 (52.3)	0	82.1 (44.6)	0
0.01-9.99 miles/week (1-59 min/week)	587	35.3 (32.4)	-1.6 (-4.7, 1.4)	82.4 (58.0)	12.2 (8.2, 16.2)***	104.9 (46.8)	11.8 (8.3, 15.3)***
≥10 miles/week (≥60 min/week)	275	42.9 (37.6)	1.9 (-2.5, 6.3)	116.6 (63.0)	35.3 (29.5, 41.0)***	128.6 (49.2)	29.7 (24.7, 34.7)***
All utility cycling							
0 miles/week (0 min/week)	10462	38.2 (37.1)	0	60.5 (51.7)	0	81.2 (44.2)	0
0.01-9.99 miles/week (1-59 min/week)	2237	42.9 (38.5)	3.7 (2.1, 5.4)***	69.6 (55.4)	5.5 (3.3, 7.7)***	89.2 (46.9)	4.4 (2.5, 6.3)***
≥10 miles/week (≥60 min/week)	647	46.7 (39.3)	6.9 (3.9, 9.8)***	99.0 (60.4)	25.0 (21.2, 28.9)***	112.1 (50.0)	20.2 (16.9, 23.6)***
Leisure-time cycling							
0 min/week	10843	37.3 (36.5)	0	60.0 (51.7)	0	80.5 (44.1)	0
1-59 min/week	1756	41.0 (35.4)	4.2 (2.3, 6.1)***	73.8 (53.9)	4.7 (2.3, 7.2)***	95.3 (45.3)	4.9 (2.8, 7.0)***
≥60 min/week	747	64.6 (46.6)	25.6 (22.9, 28.2)***	98.0 (61.5)	27.4 (23.9, 31.0)***	110.2 (52.5)	21.9 (18.9, 25.0)***
Total cycling							
0 min/week	9316	37.2 (36.6)	0	58.5 (50.8)	0	79.3 (43.6)	0
1-59 min/week	2654	39.7 (35.8)	3.2 (1.6, 4.9)***	67.0 (53.1)	2.9 (0.8, 5.0)*	88.3 (45.0)	2.6 (0.8, 4.4)*
≥60 min/week	1376	52.4 (43.6)	14.2 (12.2, 16.3)***	94.4 (60.5)	24.1 (21.3, 26.8)***	108.7 (50.7)	19.5 (17.1, 21.8)***

*p <.05, **p < .01, ***p<.001

SD: standard deviation; 95% CI: 95% confidence interval; MVPA: moderate-to-vigorous physical activity; PA: physical activity

[a]Computed as the sum of all moderate-to-vigorous leisure-time physical activity

[b]Computed as the sum of all moderate-to-vigorous physical activity across all domains (leisure-time, household, work, commute)

[c]Computed as the sum of all light and moderate-to-vigorous physical activity across all domains (leisure-time, household, work, commute)

[d]Adjusted for age, sex, education and social class

For peer review only - http://bmjopen.bmj.com/site/about/guidelines.xhtml

Appendix Part 1

Physical Activity Questions at First Health Assessment

In a typical week during the past 12 months, how many hours did you spend on each of the following activities? (Put '0' if none)

Walking, including walking to work and during leisure time
In summer ___ hours per week
In winter ___ hours per week

Cycling, including cycling to work and during leisure time
In summer ___ hours per week
In winter ___ hours per week

Other physical exercise such as keep fit, aerobics, swimming, jogging
In summer ___ hours per week
In winter ___ hours per week

For peer review only

Appendix Part 2

Table A1: Baseline characteristics (first health assessment) of the cohort (n=22,450) by all- cause, cardiovascular and cancer mortality outcomes; values are mean (SD) unless otherwise indicated

Baseline Characteristics	All-cause mortality		Cardiovascular mortality		Cancer mortality	
	Deceased (n=4380)	Survivor (n=18,070)	Deceased (n=1379)	Survivor (n=21071)	Deceased (n=1639)	Survivor (n=20811)
Follow-up years	10.5 (4.6)	16.5 (1.3)***	10.2 (4.7)	15.7 (2.9)***	10.0 (4.4)	15.8 (2.8)***
Male, N (%)	2395 (54.7)	7746 (42.9)***	782 (56.7)	9359 (44.4)***	914 (55.8)	9227 (44.3)***
Age (years)	66.1 (7.7)	56.1 (8.5)***	67.7 (6.7)	57.5 (9.0)***	63.6 (8.2)	57.6 (9.2)***
Education Level, N (%)						
No formal qualification	2049 (46.8)	5957 (33.0)	671 (48.7)	7335 (34.8)	695 (42.4)	7311 (35.1)
GCSE or equivalent	353 (8.1)	2017 (11.2)	105 (7.6)	2265 (10.8)	148 (9.0)	2222 (10.7)
A-Level or equivalent	1617 (36.9	7511 (41.6)	495 (35.9)	8633 (41.0)	654 (39.9)	8474 (40.7)
Degree or equivalent	361 (8.2)	2585 (14.3)***	108 (7.8)	2838 (13.5)***	8.7 (142)	2804 (13.5)***
Social class, N (%)						
Professional	220 (5.0)	1354 (7.5)	77 (5.6)	1497 (7.1)	79 (4.8)	1495 (7.2)
Managerial / technical	2310 (52.7)	9570 (53.0)	719 (52.1)	11161 (53.0)	834 (50.9)	54.1 (11046)
Skilled / partially skilled labour	1642 (37.5)	6568 (36.4)	502 (36.4)	7708 (36.6)	648 (39.5)	7562 (36.3)
Unskilled labour	208 (4.8)	578 (3.2)***	81 (5.9)	705 (3.4)***	78 (4.8)	708 (3.4)***
Family history of CVD, N (%)	2257 (51.5)	2123 (50.2)	756 (54.8)	10570 (50.2)**	797 (48.6)	10529 (50.6)
Family history of cancer, N (%)	1706 (39.0)	7039 (39.0)	901 (65.3)	12799 (60.8)**	709 (43.3)	38.6 (8036)***
Smoking Status, N (%)						
Current	651 (14.9)	1991 (11.0)	205 (14.9)	2437 (11.6)	281 (17.1)	2361 (11.3)
Former	2181 (49.8)	7137 (39.5)	706 (51.2)	8612 (40.9)	802 (48.9)	8516 (40.9)
Never	1548 (35.3)	8942 (49.5)***	468 (33.9)	10022 (47.6)***	556 (33.9)	9934 (47.7)***
Hypertensive Medication (yes)	1278 (29.2)	2396 (13.3)***	517 (37.5)	3157 (15.0)**	360 (22.0)	11279 (78.0)***
Lipid Medication (yes)	58 (1.6)	191 (1.1)	28 (2.0)	221 (1.1)**	15 (0.9)	234 (1.1)
Total energy intake (kj/day)	8736.4 (2527.9)	8611.4 (2513.5)**	8744.4 (2547.8)	8628.5 (2514.6)	8809.5 (2552.9)	8621.9 (2513.4)**
Units of alcohol (units/wk)	7.1 (10.6)	7.2 (9.2)	6.7 (10.0)	7.2 (9.5)*	8.0 (11.5)	7.1 (7.0)

*p < 0.05; **p < 0.005; ***p < 0.001

For peer review only

Table A2: Descriptive characteristics of participants [N (%)] at second health assessment (n=13346) by cycling (yes, no)

Baseline Characteristics	Commuter Cycling			All Utility Cycling			Leisure-time Cycling		
	None	Some	OR (95% CI)	None	Some	OR (95% CI)	None	Some	OR (95% CI)
Sex									
Male	5395 (93.0)	405 (7.0)	1	4424 (76.3)	1376 (23.7)	1	4555 (78.5)	1245 (21.5)	1
Female	7089 (93.9)	457 (6.1)	0.77 (0.66, 0.89)	6038 (80.19)	1508 (20.0)	0.83 (0.76, 0.91)	5858 (83.3)	1258 (16.7)	0.63 (0.56, 0.70)
Age (yrs)									
40 – 54	3138 (87.3)	456 (12.7)	1	2624 (73.07)	970 (27.0)	1	2534 (70.5)	1060 (29.5)	1
55 – 64	4253 (92.9)	325 (7.1)	0.50 (0.42, 0.58)	3524 (77.0)	1054 (23.0)	0.94 (0.84, 1.04)	3651 (79.8)	927 (20.3)	0.63 (0.25, 0.34)
≥65	5093 (98.4)	81 (1.6)	0.10 (0.08, 0.13)	4314 (83.4)	860 (16.6)	0.90 (0.79, 1.03)	4658 (90.0)	516 (10.0)	0.29 (0.25, 0.34)
Education Level									
Degree or equivalent	1807 (92.7)	143 (7.3)	1	1487 (76.3)	463 (23.7)	1	1493 (76.6)	457 (23.4)	1
A-Level or equivalent	5237 (93.8)	349 (6.3)	0.81 (0.66, 1.00)	4404 (78.8)	1182 (21.2)	0.90 (0.79, 1.03)	4440 (79.5)	1146 (20.5)	0.94 (0.82, 1.07)
GCSE or equivalent	1394 (93.9)	91 (6.1)	0.78 (0.59, 1.00)	1196 (80.5)	289 (19.5)	0.82 (0.69, 0.98)	1196 (80.5)	289 (19.5)	0.86 (0.72, 1.03)
No formal qualification	4046 (93.6)	279 (6.5)	1.01 (0.79, 1.29)	3375 (78.0)	950 (22.0)	1.05 (0.91, 1.22)	3714 (85.9)	611 (14.1)	0.75 (0.64, 0.88)
Social Class									
Professional	932 (93.1)	69 (6.9)	1	764 (76.3)	237 (23.7)	1	775 (77.4)	226 (22.6)	1
Managerial / technical	6990 (95.2)	350 (4.8)	0.72 (0.54, 0.95)	5927 (80.8)	1413 (19.3)	0.78 (0.66, 0.93)	5973 (81.4)	1357 (18.6)	0.85 (0.72, 1.05)
Skilled / partially skilled labour	4147 (91.2)	400 (8.8)	1.36 (1.01, 1.82)	3438 (75.6)	1109 (24.4)	1.03 (0.87, 1.23)	3708 (81.6)	839 (18.5)	0.86 (0.72, 1.04)
Unskilled labour	415 (90.6)	43 (9.4)	1.60 (1.05, 2.45)	333 (72.7)	125 (27.3)	1.25 (0.96, 1.63)	387 (84.5)	71 (15.5)	0.79 (0.58, 1.08)
Paid Employment									
Yes	6306 (88.0)	6178 (100.0)	n/a	5148 (71.8)	2020 (28.2)	1	5118 (76.0)	1617 (24.0)	1
No	862 (12.0)	0 (0)		5314 (86.0)	864 (14.0)	2.33 (2.09, 2.60)	5037 (86.9)	757 (13.1)	1.14 (1.02, 1.28)

For peer review only

Table A3: Associations between demographic characteristics of participants at the first health assessment (n=22,450) and all-cause, cardiovascular disease and cancer mortality

Demographic characteristics		N (%)	All-cause mortality		CVD mortality		Cancer mortality	
			No. deaths	Crude mortality rate (95%CI)	No. deaths	Crude mortality rate (95%CI)	No. deaths	Crude mortality rate (95%CI)
Sex	Male	10,141 (45.2)	2395	1.0	782	1.0	914	1.0
	Female	12,309 (54.8)	1985	0.62 (0.58, 0.66)	597	0.61 (0.55, 0.68)	725	0.63 (0.57, 0.70
Age (years)	40 – 54	9043 (40.3)	462	1.0	90	1.0	270	1.0
	55 – 64	6936 (30.9)	1044	3.29 (2.94, 3.7)	249	3.7 (2.9, 4.7)	537	2.7 (2.3, 3.2)
	≥65	6471 (28.8)	2874	14.8 (13.4, 16.5)	1040	19.0 (15.3, 23.7)	832	4.8 (4.2, 5.5)
Education Level	Degree or equivalent	8006 (35.7)	8006	1.0	671	1.0	695	1.0
	A-level	2370 (10.6)	2370	0.51 (0.45, 0.58)	105	0.51 (0.41, 0.63)	148	0.70 (0.58, 0.84)
	GCSE	9128 (40.7)	9128	0.63 (0.58, 0.67)	495	0.63 (0.56, 0.71)	654	0.81 (0.73, 0.91)
	No formal qualification	2946 (13.1)	2946	0.41 (0.36, 0.46)	108	0.42 (0.34, 0.10)	142	0.53 (0.44, 0.64)
Social Class	Professional	1574 (7.0)	220	1.0	77	1.0	79	1.0
	Managerial / technical	11880 (52.9)	2310	1.49 (1.28, 1.73)	719	1.25 (0.98, 1.59)	834	1.42 (1.13, 1.81)
	Skilled / partially skilled labour	8210 (36.6)	1642	1.54 (1.32, 1.79)	502	1.27 (0.99, 1.62)	648	1.62 (1.28, 2.06)
	Unskilled labour	786 (3.5)	208	2.21 (1.79, 2.74)	81	2.23 (1.61, 3.09)	78	2.08 (1.51, 2.89)
Paid employment	No	10958 (48.8)	3421	1.0	1161	1.0	1125	1.0
	Yes	11492 (51.2)	959	0.20 (0.19, 0.22)	218	0.16 (0.14, 0.19)	514	0.41 (0.37-0.46)

*p <.05, **p < .01, ***p<.001

CVD: Cardiovascular disease; 95% CI: 95% Confidence intervals

For peer review only

STROBE 2007 (v4) Statement—Checklist of items that should be included in reports of *cohort studies*

Section/Topic	Item #	Recommendation	Reported on page #
**Title and abstract**	1	(*a*) Indicate the study's design with a commonly used term in the title or the abstract	1
		(*b*) Provide in the abstract an informative and balanced summary of what was done and what was found	2
**Introduction**			
Background/rationale	2	Explain the scientific background and rationale for the investigation being reported	4
Objectives	3	State specific objectives, including any prespecified hypotheses	4
**Methods**			
Study design	4	Present key elements of study design early in the paper	5
Setting	5	Describe the setting, locations, and relevant dates, including periods of recruitment, exposure, follow-up, and data collection	5
Participants	6	(*a*) Give the eligibility criteria, and the sources and methods of selection of participants. Describe methods of follow-up	5
		(*b*) For matched studies, give matching criteria and number of exposed and unexposed	n/a
Variables	7	Clearly define all outcomes, exposures, predictors, potential confounders, and effect modifiers. Give diagnostic criteria, if applicable	5-6, 13
Data sources/ measurement	8*	For each variable of interest, give sources of data and details of methods of assessment (measurement). Describe comparability of assessment methods if there is more than one group	5-6, 13
Bias	9	Describe any efforts to address potential sources of bias	5-6
Study size	10	Explain how the study size was arrived at	5
Quantitative variables	11	Explain how quantitative variables were handled in the analyses. If applicable, describe which groupings were chosen and why	6-7
Statistical methods	12	(*a*) Describe all statistical methods, including those used to control for confounding	6-7
		(*b*) Describe any methods used to examine subgroups and interactions	n/a
		(*c*) Explain how missing data were addressed	5
		(*d*) If applicable, explain how loss to follow-up was addressed	n/a
		(*e*) Describe any sensitivity analyses	6-7
**Results**			

For peer review only

Participants	13*	(a) Report numbers of individuals at each stage of study—eg numbers potentially eligible, examined for eligibility, confirmed eligible, included in the study, completing follow-up, and analysed	5
		(b) Give reasons for non-participation at each stage	5
		(c) Consider use of a flow diagram	n/a
Descriptive data	14*	(a) Give characteristics of study participants (eg demographic, clinical, social) and information on exposures and potential confounders	5, 7, 14
		(b) Indicate number of participants with missing data for each variable of interest	5
		(c) Summarise follow-up time (eg, average and total amount)	5
Outcome data	15*	Report numbers of outcome events or summary measures over time	6
Main results	16	(*a*) Give unadjusted estimates and, if applicable, confounder-adjusted estimates and their precision (eg, 95% confidence interval). Make clear which confounders were adjusted for and why they were included	15, 16
		(*b*) Report category boundaries when continuous variables were categorized	n/a
		(*c*) If relevant, consider translating estimates of relative risk into absolute risk for a meaningful time period	n/a
Other analyses	17	Report other analyses done—eg analyses of subgroups and interactions, and sensitivity analyses	7
**Discussion**			
Key results	18	Summarise key results with reference to study objectives	8-9
**Limitations**			
Interpretation	20	Give a cautious overall interpretation of results considering objectives, limitations, multiplicity of analyses, results from similar studies, and other relevant evidence	9
Generalisability	21	Discuss the generalisability (external validity) of the study results	9
**Other information**			
Funding	22	Give the source of funding and the role of the funders for the present study and, if applicable, for the original study on which the present article is based	10

*Give information separately for cases and controls in case-control studies and, if applicable, for exposed and unexposed groups in cohort and cross-sectional studies.

Note: An Explanation and Elaboration article discusses each checklist item and gives methodological background and published examples of transparent reporting. The STROBE checklist is best used in conjunction with this article (freely available on the Web sites of PLoS Medicine at http://www.plosmedicine.org/, Annals of Internal Medicine at http://www.annals.org/, and Epidemiology at http://www.epidem.com/). Information on the STROBE Initiative is available at www.strobe-statement.org.

For peer review only

The association of cycling with all-cause, cardiovascular and cancer mortality: findings from the population based EPIC-Norfolk cohort

Journal:	*BMJ Open*
Manuscript ID:	bmjopen-2013-003797.R1
Article Type:	Research
Date Submitted by the Author:	09-Oct-2013
Complete List of Authors:	Sahlqvist, Shannon; Deakin University, Centre for Physical Activity and Nutrition Research (C-PAN); Institute of Public Health, UKCRC Centre for Diet and Activity Research<br>Goodman, Anna; London School of Hygiene and Tropical Medicine, Faculty of Epidemiology and Population Health<br>Simmons, Rebecca; Medical Research Council, Epidemiology Unit<br>Khaw, KayTee; University of Cambridge, Department of Gerontology in Clinical Medicine<br>Cavill, Nick; Cavill Associates,<br>Foster, Charles; University of Oxford, Department of Public Health<br>Luben, Robert; University of Cambridge, Department of Public Health and Primary Care, Institute of Public Health, School of Clinical Medicine<br>Wareham, Nicholas; Medical Research Council, Epidemiology Unit<br>Ogilvie, David; Medical Research Council, Epidemiology Unit
**Primary Subject Heading**:	Epidemiology
Secondary Subject Heading:	Epidemiology, Public health, Sports and exercise medicine
Keywords:	active travel, active commuting, physical activity

SCHOLARONE™ Manuscripts

For peer review only - http://bmjopen.bmj.com/site/about/guidelines.xhtml

The association of cycling with all-cause, cardiovascular and cancer mortality: findings from the population based EPIC-Norfolk cohort

Shannon Sahlqvist[1,2,3], Anna Goodman[2,4], Rebecca K. Simmons[1], Kay-Tee Khaw[5], Nick Cavill[6,7], Charlie Foster[7], Robert Luben[8,] Nicholas J. Wareham[1,2] & David Ogilvie[1,2]

[1]Medical Research Council Epidemiology Unit, University of Cambridge, UK
[2]UKCRC Centre for Diet and Activity Research (CEDAR), University of Cambridge, UK
[3]Centre for Physical Activity and Nutrition Research (C-PAN), School of Exercise and Nutrition Sciences, Deakin University, Geelong, Australia
[4]Faculty of Epidemiology and Population Health, London School of Hygiene and Tropical Medicine, London, UK
[5]Department of Gerontology in Clinical Medicine, University of Cambridge, UK
[6]Cavill Associates, Stockport, UK
[7]British Heart Foundation Health Promotion Research Group, Nuffield Department of Population Health, University of Oxford, UK
[8]Department of Public Health and Primary Care, Institute of Public Health, University of Cambridge School of Clinical Medicine, Cambridge, UK

Corresponding Author:
Shannon Sahlqvist
Centre for Physical Activity and Nutrition Research (C-PAN)
School of Exercise and Nutrition Sciences
Faculty of Health
Locked Bag 2000
Geelong Victoria 3220 Australia
Email: shannon.sahlqvist@deakin.edu.au
Telephone: 0061 3 9251 7782
Fax: 0061 2 5337 2211

Word Count: 3770
Number of pages: 23
Numbers of tables: 5

For peer review only

ABSTRACT

Objectives: To investigate associations between modest levels of total and domain-specific (commuting, other utility, recreational) cycling and mortality from all causes, cardiovascular disease and cancer.

Design: Population-based cohort study (EPIC-Norfolk).

Setting: Participants were recruited from general practices in the east of England and attended health examinations between 1993 and 1997 and again between 1998 and 2000. At the first health assessment, participants reported their average weekly duration of cycling for all purposes using a simple measure of physical activity. At the second health assessment, participants reported a more detailed breakdown of their weekly cycling behaviour using the EPAQ2 physical activity questionnaire.

Participants: Adults aged 40 – 79 years at the first health assessment.

Primary Outcome Measure: All participants were followed for mortality (all-cause, cardiovascular and cancer) until March 2011.

Results: There were 22,450 participants with complete data at the first health assessment, of whom 4,398 died during follow-up; and 13,346 participants with complete data at the second health assessment, of whom 1,670 died during follow-up. Preliminary analyses using exposure data from the first health assessment showed that cycling for at least 60 min/week in total was associated with a 9% reduced risk of all-cause mortality (adjusted hazard ratio 0.91, 95%CI 0.84, 0.99). Using the more precise measures of cycling available from the second health assessment, all types of cycling were associated with greater total moderate-to-vigorous physical activity, however there was little evidence of an association between overall or domain-specific cycling and mortality.

Conclusions: Cycling, in particular for utility purposes, was associated with greater moderate-to-vigorous and total physical activity. While this study provides tentative evidence that modest levels of cycling may reduce the risk of mortality, further research is required to confirm how much cycling is sufficient to induce health benefits.

Keywords: active travel, physical activity, commuting

For peer review only

For peer review only - http://bmjopen.bmj.com/site/about/guidelines.xhtml

ARTICLE SUMMARY

Article Focus

Cycling, particularly for transport, is promoted as a way of increasing regular physical activity among adults. Longitudinal studies have demonstrated associations between utility cycling and reduced mortality. However, these associations have been reported only for the highest exposure groups reporting substantial volumes of cycling (i.e. ≥180 min/wk).

We examined associations between mortality and lower volumes of total, and domain-specific, cycling in a population of UK adults.

Key Message

In this population with relatively low levels of cycling, preliminary analyses using a single item to measure total cycling revealed that cycling for as little as 60 min/week in total was associated with a reduced risk of all-cause mortality.

By contrast, in more substantive analyses using a detailed breakdown of cycling behaviour, neither total nor domain-specific cycling were associated with a reduced risk of mortality.

Our results provide tentative support for the hypothesis that modest 'doses' of cycling *may* reduce mortality risk and do not suggest any evidence of an adverse effect. Given that we also demonstrated that cycling is associated with the accumulation of greater total physical activity these findings contribute to the growing environmental, social and public health case for promoting cycling in individuals and populations.

Strengths and Limitations of this Study

Strengths of this study include its prospective design, the inclusion of a large heterogeneous population of men and women and the long follow-up. Further, this study used detailed measures of cycling and overall physical activity to examine associations between the various domains of cycling and mortality.

Due to the low average levels of cycling we were not able to examine the specific effects of a higher 'dose' of cycling, and the analyses were underpowered to examine sex differences in the associations between cycling and mortality.

For peer review only

For peer review only - http://bmjopen.bmj.com/site/about/guidelines.xhtml

INTRODUCTION

Promoting cycling as an alternative to motorised transport would result in reduced carbon emissions, traffic congestion and noise pollution while providing people with an opportunity to integrate regular physical activity into their lives.[1 2] As such, there is increasing policy interest in quantifying the health benefits of cycling so that they can be accurately modelled in the economic appraisal of proposed policies and interventions in the transport and health sectors.[3 4] One such tool developed by the World Health Organisation (Health Economic Assessment Tool; HEAT) estimates the economic value of a reduction in mortality as a consequence of population increases in cycling.[5] It does so by assuming a linear dose-response relationship between cycling and mortality and that any increase in cycling is in addition to other physical activity.

HEAT model estimates are dependent on the use of a relative risk estimate from a single study of Danish adults. The study reported a 28% reduction in all-cause mortality in adults who cycled to and from work compared with those who did not, even after controlling for other physical activity.[6] Similarly, an inverse association between transport (utility) cycling more generally and all-cause and cancer mortality has been reported in a cohort of Chinese women.[7] These findings are likely to reflect, in part, the fact that utility cycling translates into greater overall physical activity.[8,9]

While these studies suggest substantial health benefit associated with utility cycling, an examination of the benefits of recreational cycling would also be valuable to enable more informed policy recommendations on which type of cycling to promote.

Furthermore, it is possible that the findings from these studies reflect, at least to some extent, residual confounding from 'other' physical activity. In particular, the Danish study controlled for recreational physical activity using responses to a single item which asked participants to select from one of four options ranging from 'you are almost entirely sedentary or perform light physical activity less than two hours per week' to 'you perform highly vigorous physical activity more than four hours per week or regular exercise or competitive sports several times per week'.[6] The extent to which responses to this item were independent of those regarding commuter cycling was not reported.

In addition, in the two prior studies which reported associations between utility cycling and mortality, the time spent cycling for transport in the exposed groups was substantial, reflecting the relatively high levels of cycling in those countries. For example, in the Danish study, those who commuted by bike spent an average of 180 min/week doing so.[6] In the study of Chinese women, 19% cycled for up to 3.4 Metabolic Equivalents (MET).hr/day while a further 5% cycled for greater than 3.5 MET.hr/day, equivalent to approximately 350 min/week.[7] Few studies have examined associations between cycling and mortality in populations such as that of the UK, which have a low prevalence of utility cycling by international standards. One previous study of adults in the EPIC-Norfolk cohort found no significant association between commuter cycling and either cardiovascular or all-cause mortality.[10] These null findings may partly reflect the cut points used to define cycling categories: the cut point for the highest category was 30 min/week, which may be an insufficient 'dose' to induce health benefits. It is also possible that the relatively short duration of follow-up (seven years) and the small number of deaths in the cohort limited the power of the study to detect effects.

Building on these previous analyses of EPIC-Norfolk cohort data, this paper aims to investigate more comprehensively the mortality benefits of cycling. First, we use a simple pragmatic measure of physical activity to examine associations between total cycling and all-cause, cardiovascular and cancer mortality over 15 years. Second, using a more detailed, disaggregate measure of physical activity which provides more accurate estimates of domain-specific cycling (commuting, all utility, and recreational) for a subset of our sample, we explore whether this association is driven by particular domains of cycling (e.g. utility vs. recreational). Finally, to help explain any associations between domain-specific cycling and mortality, we examine associations between these domains of cycling and total physical activity.

For peer review only - http://bmjopen.bmj.com/site/about/guidelines.xhtml

METHODS

Study design and participants

This study uses data from the EPIC-Norfolk cohort, part of the 10-country collaborative European Prospective Investigation into Cancer and Nutrition study (EPIC). Between 1993 and 1997, 25,633 adults aged 40 – 79 years were recruited from general practices in the county of Norfolk in the east of England and attended a health examination. As part of this examination, participants completed a short physical activity questionnaire which asked about time spent walking and cycling for all purposes and time spent in other exercise.[11] Between January 1998 and October 2000 , 15,519 (61%) of the original cohort attended a second health assessment, completing a more detailed questionnaire on recreational, occupational, utility and household physical activity (EPAQ2).[12]

Data from the first health assessment were used to examine the association between total cycling and cardiovascular disease, while data from the second were used to examine the association between the domains of cycling and cardiovascular disease. Full details of the study are reported elsewhere.[13]

Of the participants in the first health assessment, we excluded those with self-reported cardiovascular disease (n=1,102) or cancer (n=1,327) and those with missing data (n=784) leaving 22,450 for analysis.

Similarly, of those who returned for the second health assessment, we excluded those with self-reported cardiovascular disease (n=772) or cancer (n=1,115) and those with missing data (n=286), leaving 13,346 for analysis. All participants were followed up for mortality to 31 March 2011 (mean 15.3 years (SD=3.3) from first health assessment, mean 11.5 years (SD = 2.0) from second health assessment). The Norwich District Health Authority Ethics Committee approved the study design and all participants provided written informed consent.

Health assessments

At both health assessments participants reported their level of education (categorised as no formal qualification; GCSE or equivalent, i.e. exams normally taken at age 16; 'A' level or equivalent, i.e. exams normally taken at age 18; university degree or equivalent), paid employment status (yes, no), social class (categorised as professional, managerial/technical job, skilled/partially skilled labour, unskilled labour), smoking status (current, former, never), anti-hypertensive medication (yes, no), medication for dyslipidaemia (yes, no) and family history of cancer and cardiovascular disease (yes, no). History of myocardial infarction, stroke, and cancer were also reported. Total energy intake (kJ/day) and alcohol consumption (units/week) were derived from a validated 130-item semi-quantitative food-frequency questionnaire.[14]

Measurement of physical activity at first health assessment

Physical activity was assessed by asking participants to report, separately for winter and summer, the weekly time (in hours) spent walking and cycling (separately) to work and during leisure, and in other exercise.[11] Total cycling was calculated as the average weekly time spent in winter and summer (min/week). See Appendix, part 1.

Measurement of physical activity at second health assessment

Physical activity, including cycling, was assessed with the validated and reliable EPAQ2 questionnaire, which asks participants to recall their physical activity behaviour across the domains of household, work, recreation and commuting, over the past year.[12] Energy expenditure [MET.hr/week] was calculated using the physical activity compendium.[15] Following standard EPAQ2 data reduction rules we calculated four specific cycling measures explained in detail in Table 1. In addition, total moderate-to-vigorous physical activity was calculated as the sum of all moderate and vigorous physical activity across all domains (home, work, recreation and commuting; MET.hr/week) and recreational physical activity was computed as the sum of all moderate and vigorous activity done during recreation specifically (MET.hr/week). A copy of the questionnaire can be found at: http://www.srl.cam.ac.uk/epic/questionnaires/epaq2/epaq2.pdf.

For peer review only - http://bmjopen.bmj.com/site/about/guidelines.xhtml

[Insert Table 1 about here]

Cycling exposure

Given the highly skewed nature of the cycling data and to allow for comparisons with previous studies, we created three categories of cycling exposure: 0 min/week, 1 – 59 min/week and ≥ 60 min/week. These represent levels of cycling which we believe are realistic to achieve in countries such as the UK, which currently have low levels of utility cycling. For our measures of utility cycling from the second health assessment these categories are equivalent to: 0 miles/week; 0.01– 9.99 miles/week and ≥ 10 miles/week.

Mortality outcomes

All EPIC-Norfolk participants were flagged for death certification with the UK Office of National Statistics (ONS). Trained nosologists coded death certificates according to the ICD-9 or ICD-10. Cardiovascular death was defined as ICD 410–448 (ICD 9) or ICD I10–I79 (ICD 10) as underlying cause of death, which comprise coronary heart disease (410–414 (ICD 9) or I20–I25 (ICD 10)), stroke (430–438 (ICD 9) or I60–I69 (ICD 10)), cardiac failure (428 (ICD 9) or I50 (ICD 10)) and other vascular causes. Cancer death was defined as ICD 140-208 (ICD9) or ICD C00 – C97 (ICD10) as the underlying cause.

Statistical analysis

We used exposure data from the first health assessment to examine associations between total cycling and all-cause, cardiovascular and cancer mortality. Exposure data from the second health assessment were used to explore associations between total, and domain specific cycling and mortality and overall physical activity. First, using data collected from the first health assessment, we examined preliminary associations between total cycling and all-cause, cardiovascular and cancer morality by fitting Cox proportional hazard regression models to estimate hazard ratios and 95% confidence intervals. We first adjusted for sex, age, education level and social class (Model A) and then further adjusted for smoking status, family history of cardiovascular disease or cancer, as well as time spent walking and in other exercise. As sensitivity analyses, we ran a further two models. In the first we adjusted for weekly alcohol consumption and calorie intake; 4% (n=912) of participants had missing data for these variables. In the second, we further adjusted for medication (hypertension and dyslipidaemia) and type 2 diabetes as we thought it possible they could be mediating variables on the causal pathway between cycling and mortality. Results of these sub-group analyses did not differ substantially from those of Model B and are not presented. Models were also run after excluding participants who died within two years of follow-up (n=181) to minimise the potential effect of reverse causality. This made no substantive differences to the findings (data not presented).

We then examined the associations between the domains of cycling and mortality, again by fitting Cox proportional hazard regression models. Equivalent models to those described above were run except that Model B also controlled for all other physical activity energy expenditure (calculated as the sum of all energy expenditure in all domains of physical activity minus that of the respective cycling behaviour). To account for the potentially conservative estimates of commuting cycling undertaken when cycling was selected alongside other modes (see Table 1), by way of sensitivity analysis we applied an alternative assumption that commuter cycling was done for 30% (rather than 10%) of these journeys. Findings remained largely unchanged when using these new estimates. Again, our results were substantively unchanged after adjusting for weekly alcohol consumption and calorie intake, or after excluding the 102 participants who died within two years of follow-up (data not presented).

For all models the proportional hazard assumption was verified using Schoenfeld residuals and Kaplan-Meier plots for all three outcomes. 'For all models, we also present p-values for linear trend, calculated by entering the domains of cycling as continuous rather than categorical variables.

For peer review only - http://bmjopen.bmj.com/site/about/guidelines.xhtml

To examine whether any observed associations between cycling and mortality could be explained by differences in overall levels of physical activity, we examined associations between the domains of cycling and physical activity (total leisure-time, total moderate-to-vigorous across all domains, and total light, moderate and vigorous across all domains) by fitting linear regression models with time spent cycling (total and sub domains) as the exposure variables and time spent in (a) recreational and (b) total moderate-to-vigorous physical activity (MET.hr/week) as the outcome variables controlling for sex, age, social class and highest level of education. All analyses were conducted using STATA, version 12.0 (Stata Corp., TX, USA).

RESULTS

Participant characteristics

At the first health assessment, participants had a mean age of 58 years (SD=19) and just over half were women (55%). 24% of participants reported cycling for a mean of 165 min/week (SD=246). Socio-demographic characteristics of the cohort by cycling status (yes, no) are described in Table 2 (for further details of the baseline characteristics of the sample see Appendix, part 2, Table A1). Respondents who reported any cycling were, on average, younger and more likely to be men. Respondents with no formal qualification were also more likely to cycle compared with respondents with GCSE-level qualifications, while those in skilled or unskilled labour were more likely to cycle than professionals.

[Insert Table 2 about here]

By the second health assessment participants had a mean age of 62 (SD 9) years; just over half were women (57%). 30% (n=4030) reported any cycling. Of those who cycled, 62% (n=2808) reported cycling for recreation and 72% (n=3269) reported cycling for utility purposes with 26% (n=862) of these reporting commuting cycling. The average cyclist spent 83 min/week cycling. Those who commuted by bicycle spent an average of 61 min/week doing so, while those who cycled for recreation spent an average of 58 min/week doing so. Again, men and those who were younger were more likely to cycle. In addition to the sociodemographic associations observed in data from the first health assessment, respondents working in a managerial/technical position were less likely to cycle than professionals. Employment status also showed a strong association with cycling, probably reflecting the fact that commuting was included in the measure of cycling (see Appendix, part 2, Table A2).

Total cycling (first health assessment) and mortality

4,398 (20%) participants died during 3,425,498 person-years of follow-up (Table 3). There were 1379 (6.1%) cardiovascular deaths and 1.639 (7.3%) cancer deaths (see Appendix, Part 2, Table A3). Risk of death was associated with being men, older, and having a lower level of education and social class.

Cycling for at least 60 min/week was associated with a 9% reduction in all-cause mortality after controlling for potential confounders (HR 0.91, 95% CI 0.84, 0.99; Table 3). In the minimally adjusted model, cycling for at least 60min/week was associated with a 19% reduction in cardiovascular mortality (HR 0.81, 95% CI 0.69, 0.95), however this was no longer significant after controlling for potential confounders including time spent walking and in other exercise. Cycling was not associated with cancer mortality.

[Insert Table 3 about here]

Domains of cycling (second health assessment) and mortality

1,670 (12.5%) individuals died during 149,072 person-years of follow-up. There were 485 (3.6%) cardiovascular deaths and 700 (5.2%) cancer deaths. Again mortality rates were higher among men and older participants (data not shown). There were no significant associations between commuting cycling and all-cause, cardiovascular or cancer mortality in either the minimally adjusted (A) or the additionally adjusted (B) models (Table 4). For both all-cause and cancer mortality, however, there was suggestion of a dose-response relationship between distance

For peer review only - http://bmjopen.bmj.com/site/about/guidelines.xhtml

cycled and risk of death whereby the lowest hazard ratios were observed for the highest levels of commuting cycling, albeit not reaching statistical significance. There was no association between all utility cycling and all-cause, cardiovascular or cancer mortality in either the minimally adjusted (A) or the additionally adjusted (B) models. In minimally adjusted models, recreational cycling for less than 60 min/week was associated with a 19% (95% CI 0.66, 0.99). Further adjusted attenuated the effect. There were no significant associations between total cycling and mortality.

[Insert Table 4 about here]

Association between domains of cycling and total physical activity (second health assessment)

Total and domain-specific cycling was associated with greater levels of physical activity in an approximately dose-response relationship (Table 5). All utility, recreation and total, but not commuting, cycling were associated with greater recreational physical activity. Importantly, however, commuting cycling was not inversely associated with recreational physical activity, suggesting that adults were not cycling to and from work to compensate for a lack of recreational physical activity. The association between cycling and recreational physical activity was strongest for recreational cycling; those who spent 1 to 59 min/week cycling for pleasure participated in an additional 3 MET.hr/day of recreational physical activity (equivalent to approximately 36 min/day of moderate intensity physical activity).

All domains of cycling showed significant dose-response relationships with total moderate-to-vigorous physical activity, although the association was strongest for commuting cycling. Those who cycled for ≥60 min/week spent an additional 7.9 MET.hr/day in moderate-to-vigorous physical activity, (equivalent to 94.8 min/day of moderate intensity physical activity) compared with those who did not.

[Insert Table 5 about here]

DISCUSSION

We used data from a large population-based cohort to examine the associations between total and domain-specific cycling and mortality. Across all domains, cyclists were more likely to be younger and men, a finding that is consistent with previous studies conducted in countries that have low rates of utility cycling[16-18] but different from the pattern in a number of other European countries where men and women, and the young and old, are equally likely to cycle.[19] An important finding was that cycling, in particular commuting cycling, was associated with participation in greater levels of total physical activity. These findings support an increasing body of work which shows that active travel is done in addition to, rather than instead of, recreational physical activity.[8 9 20 21] Given the time people spend travelling, and the fact that a shift from motorised to active travel may result in environmental and economic benefit, encouraging participation in cycling appears a valuable way to increase participation in overall physical activity.

Using exposure data from the first health assessment, cycling for at least 60 min/week in total was associated with a 9% reduction in risk of all-cause mortality but was not associated with reductions in risk of cardiovascular and cancer mortality. In the absence of any directly comparable data on total cycling from other studies, these findings provide tentative evidence that modest 'doses' of cycling may be associated with a reduction in mortality risk. They are also broadly consistent with the findings of the Danish study in which a reduction in mortality risk (28%) was associated with an average quantity of cycling that was three times higher (180min/week).[6]

That being said, when using more precise measures of cycling we found no significant associations between total or domain-specific cycling and mortality. On the one hand, these differences may reflect the more precise measures of physical activity used in the second health assessment which may have not only enabled more accurate categorisation of cycling exposure, but also reduced measurement error regarding the confounding effect of 'other' physical activity. On the other hand, they could reflect a lack of power in analyses of the second health assessment data, which included fewer participants and had a shorter follow-up period.

For peer review only - http://bmjopen.bmj.com/site/about/guidelines.xhtml

Despite five additional years of follow-up and the examination of a higher 'dose' of cycling, our null findings relating to the mortality benefits of commuting and utility cycling in particular mirror those previously reported in this cohort[10] and are consistent with those of previous studies of low-cycling populations in Northern Ireland and France, which found no evidence of a reduced risk of fatal or nonfatal myocardial infarction in men who reported any walking or cycling to work compared with those who did not.[22]

They are however in contrast to the findings of the studies of Danish[6] and Chinese[7] adults and of a meta-analysis, which pooled evidence from eight studies (from five independent populations) and found that active commuting (walking and cycling) was associated with an overall 11% reduction in the risk of cardiovascular outcomes.[23] Importantly, the levels of commuting cycling reported by participants in these previous studies were substantial and in the meta-analysis, evidence of protective effects was generally limited to higher levels of active commuting.[23] The high 'doses' of utility cycling reported in previous studies are likely to be achieved when cycling journeys are taken frequently and consistently (e.g. twice daily, five days per week). It is possible that frequent short bursts of physical activity of this kind are beneficial to health in their own right, rather than simply by contributing to greater levels of total physical activity as we have shown. In support of this hypothesis, studies have demonstrated that accumulated short bouts of exercise over the day result in longer post-exercise reductions in blood pressure[24] and lower plasma triglycerides[25] than one continuous session of exercise. There is also some evidence that the intensity of cycling is important. A study of Danish adults found a significant inverse association between cycling intensity and all-cause and coronary heart disease mortality,[26] and it may be that participants in our study were not cycling at an intensity sufficient to result in health benefit. It is also possible that the differences reflect the fact that our cohort was older than the Danish and Chinese cohorts.

To further elucidate the health benefits of cycling and refine the use of tools such as HEAT that may be used to inform policy in this area, future research should aim to estimate the association between cycling and mortality independent of other physical activity, measured with as little error as possible; to extend such analyses to include morbidity endpoints such as incident cardiovascular disease and diabetes; and to clarify how much cycling is sufficient to induce health benefits by quantifying the mean quantity (and preferably intensity) of cycling in each exposure category studied and describing the shape of the dose-response relationship. In the meantime, our results suggest that even modest 'doses' of cycling *may* reduce mortality risk and do not suggest any evidence of an adverse effect, thereby contributing to the growing environmental, social and public health case for promoting cycling in individuals and populations.

Strengths and limitations

This is the first study to examine independent associations between total and domain-specific cycling and mortality. Other strengths of the study include its prospective design, the inclusion of a large heterogeneous population of men and women and its long follow-up. We adjusted our analyses of the second health assessment data for all types of physical activity as well as a range of potential demographic and behavioural confounders which strengthens the inferences made. Excluding participants with existing chronic disease and those who died within two years of follow-up enabled us to control for reverse causality. Given the population-based recruitment from a large geographical area, we believe that our findings are generalizable to middle and older aged adults. There are, however, a number of limitations. Cycling and total physical activity were assessed by self-report. The cycling exposure variables, in particular utility cycling, were derived from relatively crude measures and assumptions had to be made about frequency of cycling, distance travelled and average speed. Due to the low average levels of cycling, we were not able to examine the specific effects of a high 'dose' of cycling and the analyses were underpowered to examine sex differences in the associations which have been previously documented.[23]

Conclusions

Building on previous research that demonstrated inverse associations between high doses of utility cycling and mortality, we used data from a large, population-based cohort to examine associations between more modest

For peer review only - http://bmjopen.bmj.com/site/about/guidelines.xhtml

levels of cycling and mortality. Cycling, in particular for utility purposes, was associated with greater levels of total and moderate-to-vigorous physical activity. This was due largely to the fact that adults who cycled did not participate in less leisure-time physical activity. Despite these positive associations, there was little evidence that cycling was associated with a reduction in mortality risk. While our preliminary findings suggest that low levels of cycling are associated with a reduced risk of mortality these findings were not replicated when using more detailed measures of exposure, albeit in fewer participants who were followed up for a shorter period. Nevertheless, cycling provides an opportunity to incorporate frequent physical activity into activities of daily living, and when done as a means to get from place to place may also confer substantial environmental and economic benefits to society.

For peer review only

For peer review only - http://bmjopen.bmj.com/site/about/guidelines.xhtml

Acknowledgements: We thank Dr Søren Brage and Dr Marcel Den Hoed for their assistance in cleaning the EPAQ2 data.

Contributors: DO and CF conceived the idea of the analysis and SS and DO designed the analysis with advice from the other authors. SS analysed the data and wrote the initial draft of the manuscript with DO, AG and RKS. KTK, NW and RL contributed to the design of the EPIC-Norfolk study protocol. All authors contributed to the critical review of the design of the analysis and the critical revision of the manuscript and approved the final version.

Funding: DO is supported by the Medical Research Council (Unit Programme number MC_UP_1001/1) and by the Centre for Diet and Activity Research (CEDAR), a UKCRC Public Health Research Centre of Excellence which also hosted SS and AG. Funding from the British Heart Foundation, Economic and Social Research Council, Medical Research Council, the National Institute for Health Research, and the Welcome Trust, under the auspices of the UK Clinical Research Collaboration, is gratefully acknowledged. AG is supported by a National Institute of Health Research (NIHR) post-doctoral fellowship. The views and opinions expressed in this article are those of the authors and do not necessarily reflect those of the NIHR, the Department of Health or other study funders. The EPIC-Norfolk study is funded by the Cancer Research Campaign; the Medical Research Council, the Stroke Association, the British Heart Foundation, the Department of Health, the Europe Against Cancer Programme Commission of the European Union and Ministry of Agriculture, Fisheries and Food. The authors thank the EPIC-Norfolk staff and participants for their valuable contributions.

Competing Interests: None

Ethics Approval: The Norwich District Health Authority Ethics Committee approved the study design and all participants provided written informed consent.

Data Sharing: Statistical code are available on request from Shannon Sahlqvist at shannon.sahlqvist@deakin.edu.au. Researchers wishing to access EPIC data to replicate or extend these analyses should contact Robert Luben (robert.luben@phpc.cam.ac.uk) in the first instance.

For peer review only

REFERENCES

1. Woodcock J, Banister D, Edwards P, et al. Energy and Health 3. *Lancet* 2007;370(9592):1078-88.
2. Shephard R. Is active commuting the answer to population health? *Sports Med* 2008;38(9):751-58.
3. Jarrett J, Woodcock J, Griffiths UK, et al. Effect of increasing active travel in urban England and Wales on costs to the National Health Service. *Lancet* 2012;379:2198-205.
4. Woodcock J, Givoni M, Morgan AS. Health Impact Modelling of Active Travel Visions for England and Wales Using an Integrated Transport and Health Impact Modelling Tool (ITHIM). *Plos One* 2013;8(1):e51462.
5. Kahlmeier S, Cavill N, Dinsdale H, et al. Health economic assessment tools (HEAT) for walking and for cycling: World Health Organization, 2011.
6. Andersen L, Schnohr P, Schroll M, et al. All-cause mortality associated with physical activity during leisure time, work, sports, and cycling to work. *Arch Intern Med* 2000;160:1621-28.
7. Matthews CE, Jurj AL, Shu X, et al. Influence of exercise, walking, cycling and overall nonexercise physical activity on mortality in Chinese women. *Am J Epid* 2007;165(12):1343-50.
8. Sahlqvist S, Song Y, Ogilvie D. Is active travel associated with greater physical activity? The contribution of commuting and non-commuting active travel to total physical activity in adults. *Prev Med* 2012;55(3):206-11.
9. Sahlqvist S, Goodman A, Cooper A, et al. Change in active travel and changes in recreational and total physical activity in adults: longitudinal findings from the iConnect study. *Int J BehavNutPhys Act* 2013;10(1):28.
10. Besson H, Ekelund U, Brage S, et al. Relationship between subdomains of total physical activity and mortality. *Med Sci Sport Exer* 2008;40(11):1909-15.
11. Khaw K-T, Jakes R, Bingham S, et al. Work and leisure time physical activity assessed using a simple, pragmatic, validated questionnaire and incident cardiovascular disease and all-cause mortality in men and women: The European Prospective Investigation into Cancer in Norfolk prospective population study. *Int J Epidemiol* 2006;35(4):1034-43.
12. Wareham N, Jakes R, Rennie K, et al. Validity and repeatability of the EPIC-Norfolk Physical Activity Questionnaire. *Int J Epidemiol* 2002;31:168-74.
13. Day N, Oakes S, Luben R, et al. EPIC Norfolk: Study design and characteristics of the cohort. *Br J Cancer* 1999;80(Supplement 1):95-103.
14. Bingham S, Gill C, Welch A, et al. Validation of dieatary assessment methods in the UK arm of EPIC using weighted records, and 24-hour urinary nitrogen and potassium and serum vitamin C and carotenoids as biomarkers. *Int J Epidemiol* 1997;26:S137-S51
15. Ainsworth BE, Haskell WL, Leon A, et al. Compendium of physical activities: classification of energy costs of human physical activities. *Med Sci SportExer* 1993;25(10):1192.
16. Sahlqvist S, Heesch K. Characteristics of utility cyclists in Queensland, Australia: An examination of the association between individual, social and environmental factors and utility cycling. *J Phys Act Health* 2012;9:818-28.
17. Winters M, Friesen MC, Koehoorn M, et al. Utilitarian bicycling: A multilevel analysis of climate and personal influences. *Am J Prev Med* 2007;32(1):52-58.
18. Foster C, Panter J, Wareham N. Assessing the impact of road traffic on cycling for leisure and cycling to work. *Int J BehavNutPhys Act* 2011;8(1):61.
19. Pucher J, Buehler R. Making cycling irresistible: Lessons from The Netherlands, Denmark and Germany. *Transport Reviews* 2008;28(4):495-528.
20. Yang L, Panter J, Griffin SJ, et al. Associations between active commuting and physical activity in working adults: Cross-sectional results from the Commuting and Health in Cambridge study. *Prev Med* 2012;55(5):453-57.
21. Wanner M, Götschi T, Martin-Diener E, et al. Active Transport, Physical Activity, and Body Weight in Adults: A Systematic Review. *Am J Prev Med* 2012;42(5):493-502.
22. Wagner A, Simon C, Evans A, et al. Physical Activity and Coronary Event Incidence in Northern Ireland and France. *Circulation* 2002;105(19):2247-52.
23. Hamer M, Chida Y. Active commuting and cardiovascular risk: a meta-analytic review. *Prev Med* 2008;46:9-13.

For peer review only - http://bmjopen.bmj.com/site/about/guidelines.xhtml

24. Park S, Rink LD, Wallace JP. Accumulation of physical activity leads to a greater blood pressure reduction than a single continuous session, in prehypertension. *J Hyper* 2006;24:1761-70.
25. Altena TS, Michaelson JL, Ball SD, et al. Single sessions of intermittent and continuous exercise and postprandial lipemia. *Med Sci Spor Exer* 2004;36(8):1364-71.
26. Schnohr P, Marott JL, Jensen JS, et al. Intensity versus duration of cycling, impact on all-cause and coronary heart disease mortality: the Copenhagen City Heart Study. *Euro J Prev Cardiol* 2012;19(1):73-80.
27. Goodman A, Brand C, Ogilvie D. Associations of health, physical activity and weight status with motorised travel and transport carbon dioxide emissions: a cross-sectional, observational study. *Environ Health* 2012;11(1):52.

For peer review only

For peer review only - http://bmjopen.bmj.com/site/about/guidelines.xhtml

TABLES

Table 1: Cycling exposure measures calculated from the EPAQ2 questionnaire administered at the second health assessment

Exposure	Calculation
Commuter cycling	Respondents were asked how frequently they normally travelled to work by car, public transport, bike or on foot (response options were 'always', 'usually', 'occasionally' or 'never/rarely'). Responses were converted to fractions (always = 1, usually = 0.75, occasionally = 0.25, never / rarely = 0). Participants reported the distance between home and work and the average number of times per week they made this journey (multiplied by two to account for the return journey). When cycling was the only mode selected, total weekly distance cycled was calculated by multiplying the distance from home to work by the number of journeys made. When cycling was selected alongside other modes, the distance cycled was weighted according to the frequency of cycling relative to the frequency of the other modes reported. For example, if a respondent selected 'always' for both cycling and driving it was assumed that cycling accounted for 10%, and driving for 90% of the distance travelled. Total number of journeys was then multiplied by the weighted distance travelled (miles/week).
Non-commuting utility cycling	Respondents were asked to recall the average number of journeys they made by bicycle to get about apart from going to work for each of the following distances: 'less than 0.5 miles', '0.5 miles to 1.5 miles', '1.5 to 2.5 miles', '2.5 to 3.5 miles', '3.5 to 5.5 miles', and 'more than 5.5 miles'. Total weekly distance travelled was computed by multiplying the reported number of trips by the midpoint value of each distance category (assumed to be 0.25 for <0.5 miles and 6 for >5.5 miles). These values were then summed to provide a measure of distance travelled (miles/week).
All utility cycling	Distance travelled for non-commuting utility cycling was added to distance travelled for commuting cycling to derive a measure of total utility cycling (miles/week).
Recreational cycling	Respondents reported the average time spent 'cycling for pleasure' per session and the frequency of such sessions: 'none', 'less than once a month', 'once a month', 'two to three times a month', 'once a week', 'two to three times a week', 'four to five times a week', or 'everyday'. Average weekly cycling duration was computed by converting the frequency into a weekly numerical value (e.g. 0.5/52 for 'less than once a month' and (2.5*12)/52 for '2 to 3 times per month'). Time spent cycling (min/week) was computed by multiplying the average session duration by the average weekly frequency.
Total cycling	To enable a measure of total cycling to be derived and to allow for comparisons with previous studies the distance travelled for utility cycling was converted into an estimated duration. Based on self-report data from a recent study of UK adults, we assumed an average cycling speed of 10 miles/hour.[27] A measure of total time spent cycling (min/week) was derived by summing time spent in commuting, other utility and recreational cycling.

For peer review only

For peer review only - http://bmjopen.bmj.com/site/about/guidelines.xhtml

Table 2: Descriptive characteristics of sample [N (%)] at first heath assessment (n=22,450) by cycling (yes, no)

Characteristic	0 min/week: N (column %)	≥ 1 min/week: N (column %)	OR for any cycling (95%CI)[a]
Sex			
Man	3880 (66.9)	1920 (72.0)	1.0
Woman	5436 (33.1)	2110 (28.0)	0.92 (0.86, 0.98)
Age (yrs)			
40 – 55	2096 (58.1)	1498 (41.7)	1.0
50 – 65	3105 (67.4)	1473 (32.2)	0.72 (0.67, 0.78)
≥65	4115 (79.4)	1059 (20.5)	0.44 (0.40, 0.49)
Education Level			
Degree or equivalent	1280 (65.6)	670 (34.4)	1.0
'A' Level or equivalent	3867 (69.2)	1719 (30.8)	0.91 (0.81, 1.02)
GCSE or equivalent	1054 (71.0)	431 (29.0)	1.02 (0.95, 1.11)
No formal qualification	3115 (72.09)	1210 (28.0)	1.29 (1.15, 1.44)
Social Class			
Professional	665 (66.4)	336 (33.6)	1.0
Managerial / technical	5269 (71.8)	2071 (28.2)	0.90 (0.79, 1.02)
Skilled / partially skilled labour	3078 (67.7)	1469 (32.3)	1.15 (1.00, 1.31)
Unskilled labour	304 (66.4)	154 (33.6)	1.36 (1.08, 1.64)
Paid Employment			
No	8578 (81.0)	2080 (19.0)	1.0
Yes	8365 (72.5)	2127 (27.2)	1.05 (0.97, 1.14)

[a]Adjusted for all other variables in the table
OR = odds ratio, CI = 95% confidence interval

For peer review only

Table 3: Prospective associations over 15 years between total cycling and mortality (all-cause, cardiovascular and cancer) in 22,450 participants

Total cycling	FU yrs Mean (SD)	All-cause mortality			Cardiovascular mortality			Cancer mortality		
		Events N (%)	Hazard Ratio (95%CI)		Events N (%)	Hazard Ratio (95%CI)		Events N (%)	Hazard Ratio (95%CI)	
			Model A[a]	Model B[b]		Model A[a]	Model B[b]		Model A[a]	Model B[b]
0 min/week	15.2 (3.4)	3,686 (21.3)	1	1	1179 (6.8)	1	1	1352 (7.8)	1	1
1 – 59 min/week	15.7 (2.6)	100 (10.8)	0.86 (0.71, 1.07)	0.96 (0.78, 1.17)	25 (2.7)	0.73 (0.49, 1.08)	0.83 (0.56, 1.24)	44 (4.7)	0.91 (0.68, 1.24)	0.99 (0.73, 1.34)
≥60 min/week	15.7 (3.0)	612 (14.3)	0.86 (0.79, 0.94)**	0.91 (0.84, 0.99)*	179 (4.2)	0.81 (0.69, 0.95)*	0.87 (0.74, 1.02)	252 (5.9)	0.89 (0.77, 1.01)	0.93 (0.81, 1.06)
p for linear trend			0.02	0.06		0.04	0.09		0.20	0.23

*p <.05, **p < .01, ***p<.001; 95% CI: 95% confidence interval; FU: follow-up

[a]Adjusted for age, sex, education level and social class

[b]Further adjusted for smoking status, family history of cancer or cardiovascular disease, and other physical activity (walking and other exercise)

For peer review only

Table 4: Prospective association over 11.5 years between cycling (total and domain specific) and mortality (all-cause, cardiovascular and cancer) in 13,346 participants

	FU yrs Mean (SD)	All-cause mortality			Cardiovascular mortality			Cancer mortality		
		Events N (%)	Hazard Ratio (95%CI)		Events N (%)	Hazard Ratio (95%CI)		Events N (%)	Hazard Ratio (95%CI)	
			Model A[a]	Model B[b]		Model A[a]	Model B[b]		Model A[a]	Model B[b]
Commuting										
0 miles/week (0 min/week)	11.1 (2.0)	1630 (13.1)	1	1	474 (3.8)	1	1	679 (5.4)	1	1
0.01-9.99 miles/week (1-59 min/week)	11.5 (1.6)	29 (4.9)	0.87 (0.60, 1.26)	0.96 (0.66, 1.39)	9 (1.5)	1.09 (0.56, 2.13)	1.23 (0.63, 2.40)	16 (2.7)	0.90 (0.55, 1.49)	0.95 (0.57, 1.57)
≥10 miles/week (≥60 min/week)	11.5 (1.4)	11 (4.0)	0.80 (0.44, 1.46)	0.91 (0.50, 1.65)	2 (0.7)	0.61 (0.15, 2.47)	0.71 (0.18, 2.90)	5 (1.8)	0.66 (0.27, 1.59)	0.68 (0.28, 1.66)
p for linear trend			0.42	0.74		1.00	0.72		0.28	0.34
All Utility										
0 miles/week 0 min/week)	11.1 (2.0)	1383 (13.2)	1	1	392 (3.8)	1	1	580 (5.5)	1	1
0.01-9.99 miles/week (1-59 min/week)	11.6 (1.8)	233 (10.4)	0.90 (0.78, 1.04)	0.95 (0.83, 1.09)	75 (3.4)	1.04 (0.81, 1.34)	1.10 (0.85, 1.41)	97 (4.3)	0.85 (0.69, 1.06)	0.90 (0.72, 1.11)
≥10 miles/week (≥60 min/week)	11.4 (1.8)	54 (8.4)	1.01 (0.77, 1.33)	1.10 (0.83, 1.44)	18 (2.8)	1.30 (0.81, 2.10)	1.44 (0.89, 2.31)	23 (3.6)	0.89 (0.58, 1.35)	0.92 (0.61, 1.41)
p for linear trend			0.71	0.33		0.81	0.52		0.94	0.89
Recreational										
0 min/week	11.1 (2.1)	1483 (13.7)	1	1	438 (4.0)	1	1	608 (5.6)	1	1
1 – 59 min/week	11.4 (1.5)	104 (5.9)	0.81 (0.66, 0.99)*	0.87 (0.69, 1.04)	25 (1.4)	0.72 (0.48, 1.09)	0.75 (0.50, 1.13)	56 (3.2)	0.90 (0.68, 1.19)	0.95 (0.71, 1.25)
≥60 min/week	11.3 (1.8)	83 (11.1)	1.13 (0.90, 1.41)	1.25 (0.99, 1.55)	22 (3.0)	1.07 (0.69, 1.65)	1.19 (0.77, 1.84)	36 (4.8)	1.06 (0.75, 1.49)	1.12 (0.80, 1.58)
p for linear trend			0.12	0.05		0.48	0.32		0.46	0.35
Total cycling										
0 min/week	11.1 (2.1)	1308 (14.0)	1	1	379 (4.1)	1	1	540 (5.8)	1	1
1 – 59 min/week	11.5 (1.8)	236 (8.9)	0.87 (0.76, 1.00)*	0.90 (0.79, 1.04)	72 (2.7)	0.95 (0.74, 1.23)	0.99 (0.76, 1.23)	105 (4.0)	0.86 (0.70, 1.06)	0.90 (0.73, 1.11)
≥60 min/week	11.4 (1.7)	126 (9.2)	1.00 (0.83, 1.21)	1.11 (0.91, 1.32)	34 (2.5)	1.00 (0.70, 1.43)	1.10 (0.77, 1.57)	55 (4.0)	0.92 (0.69, 1.22)	0.98 (0.74, 1.30)
p for linear trend			0.08	0.26		0.36	0.18		0.81	0.51

*p <.05, **p < .01, ***p<.001; 95% CI: 95% confidence interval; FU: follow-up

[a]Adjusted for age, sex, education level and social class

[b]Further adjusted for smoking status, family history of cancer or cardiovascular disease, and all other physical activity

For peer review only - http://bmjopen.bmj.com/site/about/guidelines.xhtml

Table 5: Associations between time spent cycling (total and sub domains; min/week) and physical activity (MET.hr/week) in 13,346 participants

		Leisure Time PA[a] (MET.hr/week)		Total MVPA[b] (MET.hr/week)		Total PA[c] (MET.hr/week)	
	N	Mean (SD)	Regression coefficient (95% CI)[d]	Mean (SD)	Regression coefficient (95% CI)[d]	Mean (SD)	Regression coefficient (95% CI)[d]
Commuter cycling							
0 miles/week (0 min/week)	12484	39.4 (37.7)	0	61.9 (52.3)	0	82.1 (44.6)	0
0.01-9.99 miles/week (1-59 min/week)	587	35.3 (32.4)	-1.6 (-4.7, 1.4)	82.4 (58.0)	12.2 (8.2, 16.2)***	104.9 (46.8)	11.8 (8.3, 15.3)***
≥10 miles/week (≥60 min/week)	275	42.9 (37.6)	1.9 (-2.5, 6.3)	116.6 (63.0)	35.3 (29.5, 41.0)***	128.6 (49.2)	29.7 (24.7, 34.7)***
All utility cycling							
0 miles/week (0 min/week)	10462	38.2 (37.1)	0	60.5 (51.7)	0	81.2 (44.2)	0
0.01-9.99 miles/week (1-59 min/week)	2237	42.9 (38.5)	3.7 (2.1, 5.4)***	69.6 (55.4)	5.5 (3.3, 7.7)***	89.2 (46.9)	4.4 (2.5, 6.3)***
≥10 miles/week (≥60 min/week)	647	46.7 (39.3)	6.9 (3.9, 9.8)***	99.0 (60.4)	25.0 (21.2, 28.9)***	112.1 (50.0)	20.2 (16.9, 23.6)***
Leisure-time cycling							
0 min/week	10843	37.3 (36.5)	0	60.0 (51.7)	0	80.5 (44.1)	0
1-59 min/week	1756	41.0 (35.4)	4.2 (2.3, 6.1)***	73.8 (53.9)	4.7 (2.3, 7.2)***	95.3 (45.3)	4.9 (2.8, 7.0)***
≥60 min/week	747	64.6 (46.6)	25.6 (22.9, 28.2)***	98.0 (61.5)	27.4 (23.9, 31.0)***	110.2 (52.5)	21.9 (18.9, 25.0)***
Total cycling							
0 min/week	9316	37.2 (36.6)	0	58.5 (50.8)	0	79.3 (43.6)	0
1-59 min/week	2654	39.7 (35.8)	3.2 (1.6, 4.9)***	67.0 (53.1)	2.9 (0.8, 5.0)*	88.3 (45.0)	2.6 (0.8, 4.4)*
≥60 min/week	1376	52.4 (43.6)	14.2 (12.2, 16.3)***	94.4 (60.5)	24.1 (21.3, 26.8)***	108.7 (50.7)	19.5 (17.1, 21.8)***

*p <.05, **p < .01, ***p<.001

SD: standard deviation; 95% CI: 95% confidence interval; MVPA: moderate-to-vigorous physical activity; PA: physical activity

[a]Computed as the sum of all moderate-to-vigorous leisure-time physical activity

[b]Computed as the sum of all moderate-to-vigorous physical activity across all domains (leisure-time, household, work, commute)

[c]Computed as the sum of all light and moderate-to-vigorous physical activity across all domains (leisure-time, household, work, commute)

[d]Adjusted for age, sex, education and social class

For peer review only - http://bmjopen.bmj.com/site/about/guidelines.xhtml

The association of cycling with all-cause, cardiovascular and cancer mortality: findings from the population based EPIC-Norfolk cohort

Shannon Sahlqvist[1,2,3], Anna Goodman[2,4], Rebecca K. Simmons[1], Kay-Tee Khaw[5], Nick Cavill[6,7], Charlie Foster[7], Robert Luben[8], Nicholas J. Wareham[1,2] & David Ogilvie[1,2]

[1]Medical Research Council Epidemiology Unit, University of Cambridge, UK
[2]UKCRC Centre for Diet and Activity Research (CEDAR), University of Cambridge, UK
[3]Centre for Physical Activity and Nutrition Research (C-PAN), School of Exercise and Nutrition Sciences, Deakin University, Geelong, Australia
[4]Faculty of Epidemiology and Population Health, London School of Hygiene and Tropical Medicine, London, UK
[5]Department of Gerontology in Clinical Medicine, University of Cambridge, UK
[6]Cavill Associates, Stockport, UK
[7]British Heart Foundation Health Promotion Research Group, Nuffield Department of Population Health, University of Oxford, UK
[8]Department of Public Health and Primary Care, Institute of Public Health, University of Cambridge School of Clinical Medicine, Cambridge, UK

Corresponding Author:
Shannon Sahlqvist
Centre for Physical Activity and Nutrition Research (C-PAN)
School of Exercise and Nutrition Sciences
Faculty of Health
Locked Bag 2000
Geelong Victoria 3220 Australia
Email: shannon.sahlqvist@deakin.edu.au
Telephone: 0061 3 9251 7782
Fax: 0061 2 5337 2211

Word Count: 3770
Number of pages: 23
Numbers of tables: 5

For peer review only

For peer review only - http://bmjopen.bmj.com/site/about/guidelines.xhtml

ABSTRACT

Objectives: To investigate associations between modest levels of total and domain-specific (commuting, other utility, recreational) cycling and mortality from all causes, cardiovascular disease and cancer.

Design: Population-based cohort study (EPIC-Norfolk).

Setting: Participants were recruited from general practices in the east of England and attended health examinations between 1993 and 1997 and again between 1998 and 2000. At the first health assessment, participants reported their average weekly duration of cycling for all purposes using a simple measure of physical activity. At the second health assessment, participants reported a more detailed breakdown of their weekly cycling behaviour using the EPAQ2 physical activity questionnaire.

Participants: Adults aged 40 – 79 years at the first health assessment.

Primary Outcome Measure: All participants were followed for mortality (all-cause, cardiovascular and cancer) until March 2011.

Results: There were 22,450 participants with complete data at the first health assessment, of whom 4,398 died during follow-up; and 13,346 participants with complete data at the second health assessment, of whom 1,670 died during follow-up. Preliminary analyses using exposure data from the first health assessment showed that cycling for at least 60 min/week in total was associated with a 9% reduced risk of all-cause mortality (adjusted hazard ratio 0.91, 95%CI 0.84, 0.99). Using the more precise measures of cycling available from the second health assessment, all types of cycling were associated with greater total moderate-to-vigorous physical activity, however there was little evidence of an association between overall or domain-specific cycling and mortality.

Conclusions: Cycling, in particular for utility purposes, was associated with greater moderate-to-vigorous and total physical activity. While this study provides tentative evidence that modest levels of cycling may reduce the risk of mortality, further research is required to confirm how much cycling is sufficient to induce health benefits.

Keywords: active travel, physical activity, commuting

For peer review only

For peer review only - http://bmjopen.bmj.com/site/about/guidelines.xhtml

ARTICLE SUMMARY

Article Focus

Cycling, particularly for transport, is promoted as a way of increasing regular physical activity among adults. Longitudinal studies have demonstrated associations between utility cycling and reduced mortality. However, these associations have been reported only for the highest exposure groups reporting substantial volumes of cycling (i.e. ≥180 min/wk).

We examined associations between mortality and lower volumes of total, and domain-specific, cycling in a population of UK adults.

Key Message

In this population with relatively low levels of cycling, preliminary analyses using a single item to measure total cycling revealed that cycling for as little as 60 min/week in total was associated with a reduced risk of all-cause mortality.

By contrast, in more substantive analyses using a detailed breakdown of cycling behaviour, neither total nor domain-specific cycling were associated with a reduced risk of mortality.

Our results provide tentative support for the hypothesis that modest 'doses' of cycling *may* reduce mortality risk and do not suggest any evidence of an adverse effect. Given that we also demonstrated that cycling is associated with the accumulation of greater total physical activity these findings contribute to the growing environmental, social and public health case for promoting cycling in individuals and populations.

Strengths and Limitations of this Study

Strengths of this study include its prospective design, the inclusion of a large heterogeneous population of men and women and the long follow-up. Further, this study used detailed measures of cycling and overall physical activity to examine associations between the various domains of cycling and mortality.

Due to the low average levels of cycling we were not able to examine the specific effects of a higher 'dose' of cycling, and the analyses were underpowered to examine sex differences in the associations between cycling and mortality.

For peer review only - http://bmjopen.bmj.com/site/about/guidelines.xhtml

INTRODUCTION

Promoting cycling as an alternative to motorised transport would result in reduced carbon emissions, traffic congestion and noise pollution while providing people with an opportunity to integrate regular physical activity into their lives.[1 2] As such, there is increasing policy interest in quantifying the health benefits of cycling so that they can be accurately modelled in the economic appraisal of proposed policies and interventions in the transport and health sectors.[3 4] One such tool developed by the World Health Organisation (Health Economic Assessment Tool; HEAT) estimates the economic value of a reduction in mortality as a consequence of population increases in cycling.[5] It does so by assuming a linear dose-response relationship between cycling and mortality and that any increase in cycling is in addition to other physical activity.

HEAT model estimates are dependent on the use of a relative risk estimate from a single study of Danish adults. The study reported a 28% reduction in all-cause mortality in adults who cycled to and from work compared with those who did not, even after controlling for other physical activity.[6] Similarly, an inverse association between transport (utility) cycling more generally and all-cause and cancer mortality has been reported in a cohort of Chinese women.[7] These findings are likely to reflect, in part, the fact that utility cycling translates into greater overall physical activity.[8,9]

While these studies suggest substantial health benefit associated with utility cycling, an examination of the benefits of recreational cycling would also be valuable to enable more informed policy recommendations on which type of cycling to promote.

Furthermore, it is possible that the findings from these studies reflect, at least to some extent, residual confounding from 'other' physical activity. In particular, the Danish study controlled for recreational physical activity using responses to a single item which asked participants to select from one of four options ranging from 'you are almost entirely sedentary or perform light physical activity less than two hours per week' to 'you perform highly vigorous physical activity more than four hours per week or regular exercise or competitive sports several times per week'.[6] The extent to which responses to this item were independent of those regarding commuter cycling was not reported.

In addition, in the two prior studies which reported associations between utility cycling and mortality, the time spent cycling for transport in the exposed groups was substantial, reflecting the relatively high levels of cycling in those countries. For example, in the Danish study, those who commuted by bike spent an average of 180 min/week doing so.[6] In the study of Chinese women, 19% cycled for up to 3.4 Metabolic Equivalents (MET).hr/day while a further 5% cycled for greater than 3.5 MET.hr/day, equivalent to approximately 350 min/week.[7] Few studies have examined associations between cycling and mortality in populations such as that of the UK, which have a low prevalence of utility cycling by international standards. One previous study of adults in the EPIC-Norfolk cohort found no significant association between commuter cycling and either cardiovascular or all-cause mortality.[10] These null findings may partly reflect the cut points used to define cycling categories: the cut point for the highest category was 30 min/week, which may be an insufficient 'dose' to induce health benefits. It is also possible that the relatively short duration of follow-up (seven years) and the small number of deaths in the cohort limited the power of the study to detect effects.

Building on these previous analyses of EPIC-Norfolk cohort data, this paper aims to investigate more comprehensively the mortality benefits of cycling. First, we use a simple pragmatic measure of physical activity to examine associations between total cycling and all-cause, cardiovascular and cancer mortality over 15 years. Second, using a more detailed, disaggregate measure of physical activity which provides more accurate estimates of domain-specific cycling (commuting, all utility, and recreational) for a subset of our sample, we explore whether this association is driven by particular domains of cycling (e.g. utility vs. recreational). Finally, to help explain any associations between domain-specific cycling and mortality, we examine associations between these domains of cycling and total physical activity.

For peer review only - http://bmjopen.bmj.com/site/about/guidelines.xhtml

METHODS

Study design and participants

This study uses data from the EPIC-Norfolk cohort, part of the 10-country collaborative European Prospective Investigation into Cancer and Nutrition study (EPIC). Between 1993 and 1997, 25,633 adults aged 40 – 79 years were recruited from general practices in the county of Norfolk in the east of England and attended a health examination. As part of this examination, participants completed a short physical activity questionnaire which asked about time spent walking and cycling for all purposes and time spent in other exercise.[11] Between January 1998 and October 2000 , 15,519 (61%) of the original cohort attended a second health assessment, completing a more detailed questionnaire on recreational, occupational, utility and household physical activity (EPAQ2).[12]

Data from the first health assessment were used to examine the association between total cycling and cardiovascular disease, while data from the second were used to examine the association between the domains of cycling and cardiovascular disease. Full details of the study are reported elsewhere.[13]

Of the participants in the first health assessment, we excluded those with self-reported cardiovascular disease (n=1,102) or cancer (n=1,327) and those with missing data (n=784) leaving 22,450 for analysis.

Similarly, of those who returned for the second health assessment, we excluded those with self-reported cardiovascular disease (n=772) or cancer (n=1,115) and those with missing data (n=286), leaving 13,346 for analysis. All participants were followed up for mortality to 31 March 2011 (mean 15.3 years (SD=3.3) from first health assessment, mean 11.5 years (SD = 2.0) from second health assessment). The Norwich District Health Authority Ethics Committee approved the study design and all participants provided written informed consent.

Health assessments

At both health assessments participants reported their level of education (categorised as no formal qualification; GCSE or equivalent, i.e. exams normally taken at age 16; 'A' level or equivalent, i.e. exams normally taken at age 18; university degree or equivalent), paid employment status (yes, no), social class (categorised as professional, managerial/technical job, skilled/partially skilled labour, unskilled labour), smoking status (current, former, never), anti-hypertensive medication (yes, no), medication for dyslipidaemia (yes, no) and family history of cancer and cardiovascular disease (yes, no). History of myocardial infarction, stroke, and cancer were also reported. Total energy intake (kJ/day) and alcohol consumption (units/week) were derived from a validated 130-item semi-quantitative food-frequency questionnaire.[14]

Measurement of physical activity at first health assessment

Physical activity was assessed by asking participants to report, separately for winter and summer, the weekly time (in hours) spent walking and cycling (separately) to work and during leisure, and in other exercise.[11] Total cycling was calculated as the average weekly time spent in winter and summer (min/week). See Appendix, part 1.

Measurement of physical activity at second health assessment

Physical activity, including cycling, was assessed with the validated and reliable EPAQ2 questionnaire, which asks participants to recall their physical activity behaviour across the domains of household, work, recreation and commuting, over the past year.[12] Energy expenditure [MET.hr/week] was calculated using the physical activity compendium.[15] Following standard EPAQ2 data reduction rules we calculated four specific cycling measures explained in detail in Table 1. In addition, total moderate-to-vigorous physical activity was calculated as the sum of all moderate and vigorous physical activity across all domains (home, work, recreation and commuting; MET.hr/week) and recreational physical activity was computed as the sum of all moderate and vigorous activity done during recreation specifically (MET.hr/week). A copy of the questionnaire can be found at: http://www.srl.cam.ac.uk/epic/questionnaires/epaq2/epaq2.pdf.

For peer review only - http://bmjopen.bmj.com/site/about/guidelines.xhtml

[Insert Table 1 about here]

Cycling exposure

Given the highly skewed nature of the cycling data and to allow for comparisons with previous studies, we created three categories of cycling exposure: 0 min/week, 1 – 59 min/week and ≥ 60 min/week. These represent levels of cycling which we believe are realistic to achieve in countries such as the UK, which currently have low levels of utility cycling. For our measures of utility cycling from the second health assessment these categories are equivalent to: 0 miles/week; 0.01– 9.99 miles/week and ≥ 10 miles/week.

Mortality outcomes

All EPIC-Norfolk participants were flagged for death certification with the UK Office of National Statistics (ONS). Trained nosologists coded death certificates according to the ICD-9 or ICD-10. Cardiovascular death was defined as ICD 410–448 (ICD 9) or ICD I10–I79 (ICD 10) as underlying cause of death, which comprise coronary heart disease (410–414 (ICD 9) or I20–I25 (ICD 10)), stroke (430–438 (ICD 9) or I60–I69 (ICD 10)), cardiac failure (428 (ICD 9) or I50 (ICD 10)) and other vascular causes. Cancer death was defined as ICD 140-208 (ICD9) or ICD C00 – C97 (ICD10) as the underlying cause.

Statistical analysis

We used exposure data from the first health assessment to examine associations between total cycling and all-cause, cardiovascular and cancer mortality. Exposure data from the second health assessment were used to explore associations between total, and domain specific cycling and mortality and overall physical activity. First, using data collected from the first health assessment, we examined preliminary associations between total cycling and all-cause, cardiovascular and cancer morality by fitting Cox proportional hazard regression models to estimate hazard ratios and 95% confidence intervals. We first adjusted for sex, age, education level and social class (Model A) and then further adjusted for smoking status, family history of cardiovascular disease or cancer, as well as time spent walking and in other exercise. As sensitivity analyses, we ran a further two models. In the first we adjusted for weekly alcohol consumption and calorie intake; 4% (n=912) of participants had missing data for these variables. In the second, we further adjusted for medication (hypertension and dyslipidaemia) and type 2 diabetes as we thought it possible they could be mediating variables on the causal pathway between cycling and mortality. Results of these sub-group analyses did not differ substantially from those of Model B and are not presented. Models were also run after excluding participants who died within two years of follow-up (n=181) to minimise the potential effect of reverse causality. This made no substantive differences to the findings (data not presented).

We then examined the associations between the domains of cycling and mortality, again by fitting Cox proportional hazard regression models. Equivalent models to those described above were run except that Model B also controlled for all other physical activity energy expenditure (calculated as the sum of all energy expenditure in all domains of physical activity minus that of the respective cycling behaviour). To account for the potentially conservative estimates of commuting cycling undertaken when cycling was selected alongside other modes (see Table 1), by way of sensitivity analysis we applied an alternative assumption that commuter cycling was done for 30% (rather than 10%) of these journeys. Findings remained largely unchanged when using these new estimates. Again, our results were substantively unchanged after adjusting for weekly alcohol consumption and calorie intake, or after excluding the 102 participants who died within two years of follow-up (data not presented).

For all models the proportional hazard assumption was verified using Schoenfeld residuals and Kaplan-Meier plots for all three outcomes. 'For all models, we also present p-values for linear trend, calculated by entering the domains of cycling as continuous rather than categorical variables.

For peer review only - http://bmjopen.bmj.com/site/about/guidelines.xhtml

To examine whether any observed associations between cycling and mortality could be explained by differences in overall levels of physical activity, we examined associations between the domains of cycling and physical activity (total leisure-time, total moderate-to-vigorous across all domains, and total light, moderate and vigorous across all domains) by fitting linear regression models with time spent cycling (total and sub domains) as the exposure variables and time spent in (a) recreational and (b) total moderate-to-vigorous physical activity (MET.hr/week) as the outcome variables controlling for sex, age, social class and highest level of education. All analyses were conducted using STATA, version 12.0 (Stata Corp., TX, USA).

RESULTS

Participant characteristics

At the first health assessment, participants had a mean age of 58 years (SD=19) and just over half were women (55%). 24% of participants reported cycling for a mean of 165 min/week (SD=246). Socio-demographic characteristics of the cohort by cycling status (yes, no) are described in Table 2 (for further details of the baseline characteristics of the sample see Appendix, part 2, Table A1). Respondents who reported any cycling were, on average, younger and more likely to be men. Respondents with no formal qualification were also more likely to cycle compared with respondents with GCSE-level qualifications, while those in skilled or unskilled labour were more likely to cycle than professionals.

[Insert Table 2 about here]

By the second health assessment participants had a mean age of 62 (SD 9) years; just over half were women (57%). 30% (n=4030) reported any cycling. Of those who cycled, 62% (n=2808) reported cycling for recreation and 72% (n=3269) reported cycling for utility purposes with 26% (n=862) of these reporting commuting cycling. The average cyclist spent 83 min/week cycling. Those who commuted by bicycle spent an average of 61 min/week doing so, while those who cycled for recreation spent an average of 58 min/week doing so. Again, men and those who were younger were more likely to cycle. In addition to the sociodemographic associations observed in data from the first health assessment, respondents working in a managerial/technical position were less likely to cycle than professionals. Employment status also showed a strong association with cycling, probably reflecting the fact that commuting was included in the measure of cycling (see Appendix, part 2, Table A2).

Total cycling (first health assessment) and mortality

4,398 (20%) participants died during 3,425,498 person-years of follow-up (Table 3). There were 1379 (6.1%) cardiovascular deaths and 1.639 (7.3%) cancer deaths (see Appendix, Part 2, Table A3). Risk of death was associated with being men, older, and having a lower level of education and social class.

Cycling for at least 60 min/week was associated with a 9% reduction in all-cause mortality after controlling for potential confounders (HR 0.91, 95% CI 0.84, 0.99; Table 3). In the minimally adjusted model, cycling for at least 60min/week was associated with a 19% reduction in cardiovascular mortality (HR 0.81, 95% CI 0.69, 0.95), however this was no longer significant after controlling for potential confounders including time spent walking and in other exercise. Cycling was not associated with cancer mortality.

[Insert Table 3 about here]

Domains of cycling (second health assessment) and mortality

1,670 (12.5%) individuals died during 149,072 person-years of follow-up. There were 485 (3.6%) cardiovascular deaths and 700 (5.2%) cancer deaths. Again mortality rates were higher among men and older participants (data not shown). There were no significant associations between commuting cycling and all-cause, cardiovascular or cancer mortality in either the minimally adjusted (A) or the additionally adjusted (B) models (Table 4). For both all-cause and cancer mortality, however, there was suggestion of a dose-response relationship between distance

For peer review only - http://bmjopen.bmj.com/site/about/guidelines.xhtml

cycled and risk of death whereby the lowest hazard ratios were observed for the highest levels of commuting cycling, albeit not reaching statistical significance. There was no association between all utility cycling and all-cause, cardiovascular or cancer mortality in either the minimally adjusted (A) or the additionally adjusted (B) models. In minimally adjusted models, recreational cycling for less than 60 min/week was associated with a 19% (95% CI 0.66, 0.99). Further adjusted attenuated the effect. There were no significant associations between total cycling and mortality.

[Insert Table 4 about here]

Association between domains of cycling and total physical activity (second health assessment)

Total and domain-specific cycling was associated with greater levels of physical activity in an approximately dose-response relationship (Table 5). All utility, recreation and total, but not commuting, cycling were associated with greater recreational physical activity. Importantly, however, commuting cycling was not inversely associated with recreational physical activity, suggesting that adults were not cycling to and from work to compensate for a lack of recreational physical activity. The association between cycling and recreational physical activity was strongest for recreational cycling; those who spent 1 to 59 min/week cycling for pleasure participated in an additional 3 MET.hr/day of recreational physical activity (equivalent to approximately 36 min/day of moderate intensity physical activity).

All domains of cycling showed significant dose-response relationships with total moderate-to-vigorous physical activity, although the association was strongest for commuting cycling. Those who cycled for ≥60 min/week spent an additional 7.9 MET.hr/day in moderate-to-vigorous physical activity, (equivalent to 94.8 min/day of moderate intensity physical activity) compared with those who did not.

[Insert Table 5 about here]

DISCUSSION

We used data from a large population-based cohort to examine the associations between total and domain-specific cycling and mortality. Across all domains, cyclists were more likely to be younger and men, a finding that is consistent with previous studies conducted in countries that have low rates of utility cycling[16-18] but different from the pattern in a number of other European countries where men and women, and the young and old, are equally likely to cycle.[19] An important finding was that cycling, in particular commuting cycling, was associated with participation in greater levels of total physical activity. These findings support an increasing body of work which shows that active travel is done in addition to, rather than instead of, recreational physical activity.[8 9 20 21] Given the time people spend travelling, and the fact that a shift from motorised to active travel may result in environmental and economic benefit, encouraging participation in cycling appears a valuable way to increase participation in overall physical activity.

Using exposure data from the first health assessment, cycling for at least 60 min/week in total was associated with a 9% reduction in risk of all-cause mortality but was not associated with reductions in risk of cardiovascular and cancer mortality. In the absence of any directly comparable data on total cycling from other studies, these findings provide tentative evidence that modest 'doses' of cycling may be associated with a reduction in mortality risk. They are also broadly consistent with the findings of the Danish study in which a reduction in mortality risk (28%) was associated with an average quantity of cycling that was three times higher (180min/week).[6]

That being said, when using more precise measures of cycling we found no significant associations between total or domain-specific cycling and mortality. On the one hand, these differences may reflect the more precise measures of physical activity used in the second health assessment which may have not only enabled more accurate categorisation of cycling exposure, but also reduced measurement error regarding the confounding effect of 'other' physical activity. On the other hand, they could reflect a lack of power in analyses of the second health assessment data, which included fewer participants and had a shorter follow-up period.

For peer review only - http://bmjopen.bmj.com/site/about/guidelines.xhtml

Despite five additional years of follow-up and the examination of a higher 'dose' of cycling, our null findings relating to the mortality benefits of commuting and utility cycling in particular mirror those previously reported in this cohort[10] and are consistent with those of previous studies of low-cycling populations in Northern Ireland and France, which found no evidence of a reduced risk of fatal or nonfatal myocardial infarction in men who reported any walking or cycling to work compared with those who did not.[22]

They are however in contrast to the findings of the studies of Danish[6] and Chinese[7] adults and of a meta-analysis, which pooled evidence from eight studies (from five independent populations) and found that active commuting (walking and cycling) was associated with an overall 11% reduction in the risk of cardiovascular outcomes.[23] Importantly, the levels of commuting cycling reported by participants in these previous studies were substantial and in the meta-analysis, evidence of protective effects was generally limited to higher levels of active commuting.[23] The high 'doses' of utility cycling reported in previous studies are likely to be achieved when cycling journeys are taken frequently and consistently (e.g. twice daily, five days per week). It is possible that frequent short bursts of physical activity of this kind are beneficial to health in their own right, rather than simply by contributing to greater levels of total physical activity as we have shown. In support of this hypothesis, studies have demonstrated that accumulated short bouts of exercise over the day result in longer post-exercise reductions in blood pressure[24] and lower plasma triglycerides[25] than one continuous session of exercise. There is also some evidence that the intensity of cycling is important. A study of Danish adults found a significant inverse association between cycling intensity and all-cause and coronary heart disease mortality,[26] and it may be that participants in our study were not cycling at an intensity sufficient to result in health benefit. It is also possible that the differences reflect the fact that our cohort was older than the Danish and Chinese cohorts.

To further elucidate the health benefits of cycling and refine the use of tools such as HEAT that may be used to inform policy in this area, future research should aim to estimate the association between cycling and mortality independent of other physical activity, measured with as little error as possible; to extend such analyses to include morbidity endpoints such as incident cardiovascular disease and diabetes; and to clarify how much cycling is sufficient to induce health benefits by quantifying the mean quantity (and preferably intensity) of cycling in each exposure category studied and describing the shape of the dose-response relationship. In the meantime, our results suggest that even modest 'doses' of cycling *may* reduce mortality risk and do not suggest any evidence of an adverse effect, thereby contributing to the growing environmental, social and public health case for promoting cycling in individuals and populations.

Strengths and limitations

This is the first study to examine independent associations between total and domain-specific cycling and mortality. Other strengths of the study include its prospective design, the inclusion of a large heterogeneous population of men and women and its long follow-up. We adjusted our analyses of the second health assessment data for all types of physical activity as well as a range of potential demographic and behavioural confounders which strengthens the inferences made. Excluding participants with existing chronic disease and those who died within two years of follow-up enabled us to control for reverse causality. Given the population-based recruitment from a large geographical area, we believe that our findings are generalizable to middle and older aged adults. There are, however, a number of limitations. Cycling and total physical activity were assessed by self-report. The cycling exposure variables, in particular utility cycling, were derived from relatively crude measures and assumptions had to be made about frequency of cycling, distance travelled and average speed. Due to the low average levels of cycling, we were not able to examine the specific effects of a high 'dose' of cycling and the analyses were underpowered to examine sex differences in the associations which have been previously documented.[23]

Conclusions

Building on previous research that demonstrated inverse associations between high doses of utility cycling and mortality, we used data from a large, population-based cohort to examine associations between more modest

For peer review only - http://bmjopen.bmj.com/site/about/guidelines.xhtml

levels of cycling and mortality. Cycling, in particular for utility purposes, was associated with greater levels of total and moderate-to-vigorous physical activity. This was due largely to the fact that adults who cycled did not participate in less leisure-time physical activity. Despite these positive associations, there was little evidence that cycling was associated with a reduction in mortality risk. While our preliminary findings suggest that low levels of cycling are associated with a reduced risk of mortality these findings were not replicated when using more detailed measures of exposure, albeit in fewer participants who were followed up for a shorter period. Nevertheless, cycling provides an opportunity to incorporate frequent physical activity into activities of daily living, and when done as a means to get from place to place may also confer substantial environmental and economic benefits to society.

For peer review only

For peer review only - http://bmjopen.bmj.com/site/about/guidelines.xhtml

Acknowledgements: We thank Dr Søren Brage and Dr Marcel Den Hoed for their assistance in cleaning the EPAQ2 data.

Contributors: DO and CF conceived the idea of the analysis and SS and DO designed the analysis with advice from the other authors. SS analysed the data and wrote the initial draft of the manuscript with DO, AG and RKS. KTK, NW and RL contributed to the design of the EPIC-Norfolk study protocol. All authors contributed to the critical review of the design of the analysis and the critical revision of the manuscript and approved the final version.

Funding: DO is supported by the Medical Research Council (Unit Programme number MC_UP_1001/1) and by the Centre for Diet and Activity Research (CEDAR), a UKCRC Public Health Research Centre of Excellence which also hosted SS and AG. Funding from the British Heart Foundation, Economic and Social Research Council, Medical Research Council, the National Institute for Health Research, and the Welcome Trust, under the auspices of the UK Clinical Research Collaboration, is gratefully acknowledged. AG is supported by a National Institute of Health Research (NIHR) post-doctoral fellowship. The views and opinions expressed in this article are those of the authors and do not necessarily reflect those of the NIHR, the Department of Health or other study funders. The EPIC-Norfolk study is funded by the Cancer Research Campaign; the Medical Research Council, the Stroke Association, the British Heart Foundation, the Department of Health, the Europe Against Cancer Programme Commission of the European Union and Ministry of Agriculture, Fisheries and Food. The authors thank the EPIC-Norfolk staff and participants for their valuable contributions.

Competing Interests: None

Ethics Approval: The Norwich District Health Authority Ethics Committee approved the study design and all participants provided written informed consent.

For peer review only

For peer review only - http://bmjopen.bmj.com/site/about/guidelines.xhtml

REFERENCES

1. Woodcock J, Banister D, Edwards P, Prentice AM, Roberts I. Energy and Health 3. *Lancet* 2007;370(9592):1078-88.
2. Shephard R. Is active commuting the answer to population health? *Sports Med* 2008;38(9):751-58.
3. Jarrett J, Woodcock J, Griffiths UK, Chalabi Z, Edwards P, Roberts I, et al. Effect of increasing active travel in urban England and Wales on costs to the National Health Service. *Lancet* 2012;379:2198-205.
4. Woodcock J, Givoni M, Morgan AS. Health Impact Modelling of Active Travel Visions for England and Wales Using an Integrated Transport and Health Impact Modelling Tool (ITHIM). *Plos One* 2013;8(1):e51462.
5. Kahlmeier S, Cavill N, Dinsdale H, Rutter H, Götschi T, Foster C, et al. Health economic assessment tools (HEAT) for walking and for cycling: World Health Organization, 2011.
6. Andersen L, Schnohr P, Schroll M, Hein A. All-cause mortality associated with physical activity during leisure time, work, sports, and cycling to work. *Arch Intern Med* 2000;160:1621-28.
7. Matthews CE, Jurj AL, Shu X, Li H-L, Yang G, Li Q, et al. Influence of exercise, walking, cycling and overall nonexercise physical activity on mortality in Chinese women. *Am J Epid* 2007;165(12):1343-50.
8. Sahlqvist S, Song Y, Ogilvie D. Is active travel associated with greater physical activity? The contribution of commuting and non-commuting active travel to total physical activity in adults. *Prev Med* 2012;55(3):206-11.
9. Sahlqvist S, Goodman A, Cooper A, Ogilvie D, consortium. oboti. Change in active travel and changes in recreational and total physical activity in adults: longitudinal findings from the iConnect study. *Int J BehavNutPhys Act* 2013;10(1):28.
10. Besson H, Ekelund U, Brage S, Luben R, Bingham S, Khaw K-T, et al. Relationship between subdomains of total physical activity and mortality. *Med Sci Sport Exer* 2008;40(11):1909-15.
11. Khaw K-T, Jakes R, Bingham S, Welch A, Luben R, Day N, et al. Work and leisure time physical activity assessed using a simple, pragmatic, validated questionnaire and incident cardiovascular disease and all-cause mortality in men and women: The European Prospective Investigation into Cancer in Norfolk prospective population study. *Int J Epidemiol* 2006;35(4):1034-43.
12. Wareham N, Jakes R, Rennie K, Mitchell J, Hennings S, Day N. Validity and repeatability of the EPIC-Norfolk Physical Activity Questionnaire. *Int J Epidemiol* 2002;31:168-74.
13. Day N, Oakes S, Luben R, Khaw K-T, Bingham S, Welch A, et al. EPIC Norfolk: Study design and characteristics of the cohort. *Br J Cancer* 1999;80(Supplement 1):95-103.
14. Bingham S, Gill C, Welch A, Cassidy A, Runswick S, al e. Validation of dieatary assessment methods in the UK arm of EPIC using weighted records, and 24-hour urinary nitrogen and potassium and serum vitamin C and carotenoids as biomarkers. *Int J Epidemiol* 1997;26:S137-S51
15. Ainsworth BE, Haskell WL, Leon A, Jacobs DJ, Montoye H, Sallis J, et al. Compendium of physical activities: classification of energy costs of human physical activities. *Med Sci SportExer* 1993;25(10):1192.
16. Sahlqvist S, Heesch K. Characteristics of utility cyclists in Queensland, Australia: An examination of the association between individual, social and environmental factors and utility cycling. *J Phys Act Health* 2012;9:818-28.
17. Winters M, Friesen MC, Koehoorn M, Teschke K. Utilitarian bicycling: A multilevel analysis of climate and personal influences. *Am J Prev Med* 2007;32(1):52-58.
18. Foster C, Panter J, Wareham N. Assessing the impact of road traffic on cycling for leisure and cycling to work. *Int J BehavNutPhys Act* 2011;8(1):61.
19. Pucher J, Buehler R. Making cycling irresistible: Lessons from The Netherlands, Denmark and Germany. *Transport Reviews* 2008;28(4):495-528.
20. Yang L, Panter J, Griffin SJ, Ogilvie D. Associations between active commuting and physical activity in working adults: Cross-sectional results from the Commuting and Health in Cambridge study. *Prev Med* 2012;55(5):453-57.

For peer review only

For peer review only - http://bmjopen.bmj.com/site/about/guidelines.xhtml

21. Wanner M, Götschi T, Martin-Diener E, Kahlmeier S, Martin BW. Active Transport, Physical Activity, and Body Weight in Adults: A Systematic Review. *Am J Prev Med* 2012;42(5):493-502.
22. Wagner A, Simon C, Evans A, Ferrières J, Montaye M, Ducimetière P, et al. Physical Activity and Coronary Event Incidence in Northern Ireland and France. *Circulation* 2002;105(19):2247-52.
23. Hamer M, Chida Y. Active commuting and cardiovascular risk: a meta-analytic review. *Prev Med* 2008;46:9-13.
24. Park S, Rink LD, Wallace JP. Accumulation of physical activity leads to a greater blood pressure reduction than a single continuous session, in prehypertension. *J Hyper* 2006;24:1761-70.
25. Altena TS, Michaelson JL, Ball SD, Thomas TR. Single sessions of intermittent and continuous exercise and postprandial lipemia. *Med Sci Spor Exer* 2004;36(8):1364-71.
26. Schnohr P, Marott JL, Jensen JS, Jensen GB. Intensity versus duration of cycling, impact on all-cause and coronary heart disease mortality: the Copenhagen City Heart Study. *Euro J Prev Cardiol* 2012;19(1):73-80.
27. Goodman A, Brand C, Ogilvie D. Associations of health, physical activity and weight status with motorised travel and transport carbon dioxide emissions: a cross-sectional, observational study. *Environ Health* 2012;11(1):52.

For peer review only

For peer review only - http://bmjopen.bmj.com/site/about/guidelines.xhtml

TABLES

Table 1: Cycling exposure measures calculated from the EPAQ2 questionnaire administered at the second health assessment

Exposure	Calculation
Commuter cycling	Respondents were asked how frequently they normally travelled to work by car, public transport, bike or on foot (response options were ‘always’, ‘usually’, ‘occasionally’ or ‘never/rarely’). Responses were converted to fractions (always = 1, usually = 0.75, occasionally = 0.25, never / rarely = 0). Participants reported the distance between home and work and the average number of times per week they made this journey (multiplied by two to account for the return journey). When cycling was the only mode selected, total weekly distance cycled was calculated by multiplying the distance from home to work by the number of journeys made. When cycling was selected alongside other modes, the distance cycled was weighted according to the frequency of cycling relative to the frequency of the other modes reported. For example, if a respondent selected ‘always’ for both cycling and driving it was assumed that cycling accounted for 10%, and driving for 90% of the distance travelled. Total number of journeys was then multiplied by the weighted distance travelled (miles/week).
Non-commuting utility cycling	Respondents were asked to recall the average number of journeys they made by bicycle to get about apart from going to work for each of the following distances: ‘less than 0.5 miles’, ‘0.5 miles to 1.5 miles’, ‘1.5 to 2.5 miles’, ‘2.5 to 3.5 miles’, ‘3.5 to 5.5 miles’, and ‘more than 5.5 miles’. Total weekly distance travelled was computed by multiplying the reported number of trips by the midpoint value of each distance category (assumed to be 0.25 for <0.5 miles and 6 for >5.5 miles). These values were then summed to provide a measure of distance travelled (miles/week).
All utility cycling	Distance travelled for non-commuting utility cycling was added to distance travelled for commuting cycling to derive a measure of total utility cycling (miles/week).
Recreational cycling	Respondents reported the average time spent ‘cycling for pleasure’ per session and the frequency of such sessions: ‘none’, ‘less than once a month’, ‘once a month’, ‘two to three times a month’, ‘once a week’, ‘two to three times a week’, ‘four to five times a week’, or ‘everyday’. Average weekly cycling duration was computed by converting the frequency into a weekly numerical value (e.g. 0.5/52 for ‘less than once a month’ and (2.5*12)/52 for ‘2 to 3 times per month’). Time spent cycling (min/week) was computed by multiplying the average session duration by the average weekly frequency.
Total cycling	To enable a measure of total cycling to be derived and to allow for comparisons with previous studies the distance travelled for utility cycling was converted into an estimated duration. Based on self-report data from a recent study of UK adults, we assumed an average cycling speed of 10 miles/hour.[27] A measure of total time spent cycling (min/week) was derived by summing time spent in commuting, other utility and recreational cycling.

For peer review only

Table 2: Descriptive characteristics of sample [N (%)] at first heath assessment (n=22,450) by cycling (yes, no)

Characteristic	0 min/week: N (column %)	≥ 1 min/week: N (column %)	OR for any cycling (95%CI)[a]
Sex			
Man	3880 (66.9)	1920 (72.0)	1.0
Woman	5436 (33.1)	2110 (28.0)	0.92 (0.86, 0.98)
Age (yrs)			
40 – 55	2096 (58.1)	1498 (41.7)	1.0
50 – 65	3105 (67.4)	1473 (32.2)	0.72 (0.67, 0.78)
≥65	4115 (79.4)	1059 (20.5)	0.44 (0.40, 0.49)
Education Level			
Degree or equivalent	1280 (65.6)	670 (34.4)	1.0
'A' Level or equivalent	3867 (69.2)	1719 (30.8)	0.91 (0.81, 1.02)
GCSE or equivalent	1054 (71.0)	431 (29.0)	1.02 (0.95, 1.11)
No formal qualification	3115 (72.09)	1210 (28.0)	1.29 (1.15, 1.44)
Social Class			
Professional	665 (66.4)	336 (33.6)	1.0
Managerial / technical	5269 (71.8)	2071 (28.2)	0.90 (0.79, 1.02)
Skilled / partially skilled labour	3078 (67.7)	1469 (32.3)	1.15 (1.00, 1.31)
Unskilled labour	304 (66.4)	154 (33.6)	1.36 (1.08, 1.64)
Paid Employment			
No	8578 (81.0)	2080 (19.0)	1.0
Yes	8365 (72.5)	2127 (27.2)	1.05 (0.97, 1.14)

[a]Adjusted for all other variables in the table
OR = odds ratio, CI = 95% confidence interval

For peer review only

For peer review only - http://bmjopen.bmj.com/site/about/guidelines.xhtml

Table 3: Prospective associations over 15 years between total cycling and mortality (all-cause, cardiovascular and cancer) in 22,450 participants

Total cycling	FU yrs Mean (SD)	All-cause mortality			Cardiovascular mortality			Cancer mortality		
		Events N (%)	Hazard Ratio (95%CI)		Events N (%)	Hazard Ratio (95%CI)		Events N (%)	Hazard Ratio (95%CI)	
			Model A[a]	Model B[b]		Model A[a]	Model B[b]		Model A[a]	Model B[b]
0 min/week	15.2 (3.4)	3,686 (21.3)	1	1	1179 (6.8)	1	1	1352 (7.8)	1	1
1 – 59 min/week	15.7 (2.6)	100 (10.8)	0.86 (0.71, 1.07)	0.96 (0.78, 1.17)	25 (2.7)	0.73 (0.49, 1.08)	0.83 (0.56, 1.24)	44 (4.7)	0.91 (0.68, 1.24)	0.99 (0.73, 1.34)
≥60 min/week	15.7 (3.0)	612 (14.3)	0.86 (0.79, 0.94)**	0.91 (0.84, 0.99)*	179 (4.2)	0.81 (0.69, 0.95)*	0.87 (0.74, 1.02)	252 (5.9)	0.89 (0.77, 1.01)	0.93 (0.81, 1.06)
p for linear trend			0.02	0.06		0.04	0.09		0.20	0.23

*p <.05, **p < .01, ***p<.001; 95% CI: 95% confidence interval; FU: follow-up
[a]Adjusted for age, sex, education level and social class
[b]Further adjusted for smoking status, family history of cancer or cardiovascular disease, and other physical activity (walking and other exercise)

16

For peer review only

Table 4: Prospective association over 11.5 years between cycling (total and domain specific) and mortality (all-cause, cardiovascular and cancer) in 13,346 participants

	FU yrs Mean (SD)	All-cause mortality			Cardiovascular mortality			Cancer mortality		
		Events N (%)	Hazard Ratio (95%CI)		Events N (%)	Hazard Ratio (95%CI)		Events N (%)	Hazard Ratio (95%CI)	
			Model A[a]	Model B[b]		Model A[a]	Model B[b]		Model A[a]	Model B[b]
Commuting										
0 miles/week (0 min/week)	11.1 (2.0)	1630 (13.1)	1	1	474 (3.8)	1	1	679 (5.4)	1	1
0.01-9.99 miles/week (1-59 min/week)	11.5 (1.6)	29 (4.9)	0.87 (0.60, 1.26)	0.96 (0.66, 1.39)	9 (1.5)	1.09 (0.56, 2.13)	1.23 (0.63, 2.40)	16 (2.7)	0.90 (0.55, 1.49)	0.95 (0.57, 1.57)
≥10 miles/week (≥60 min/week)	11.5 (1.4)	11 (4.0)	0.80 (0.44, 1.46)	0.91 (0.50, 1.65)	2 (0.7)	0.61 (0.15, 2.47)	0.71 (0.18, 2.90)	5 (1.8)	0.66 (0.27, 1.59)	0.68 (0.28, 1.66)
p for linear trend			0.42	0.74		1.00	0.72		0.28	0.34
All Utility										
0 miles/week 0 min/week)	11.1 (2.0)	1383 (13.2)	1	1	392 (3.8)	1	1	580 (5.5)	1	1
0.01-9.99 miles/week (1-59 min/week)	11.6 (1.8)	233 (10.4)	0.90 (0.78, 1.04)	0.95 (0.83, 1.09)	75 (3.4)	1.04 (0.81, 1.34)	1.10 (0.85, 1.41)	97 (4.3)	0.85 (0.69, 1.06)	0.90 (0.72, 1.11)
≥10 miles/week (≥60 min/week)	11.4 (1.8)	54 (8.4)	1.01 (0.77, 1.33)	1.10 (0.83, 1.44)	18 (2.8)	1.30 (0.81, 2.10)	1.44 (0.89, 2.31)	23 (3.6)	0.89 (0.58, 1.35)	0.92 (0.61, 1.41)
p for linear trend			0.71	0.33		0.81	0.52		0.94	0.89
Recreational										
0 min/week	11.1 (2.1)	1483 (13.7)	1	1	438 (4.0)	1	1	608 (5.6)	1	1
1 – 59 min/week	11.4 (1.5)	104 (5.9)	0.81 (0.66, 0.99)*	0.87 (0.69, 1.04)	25 (1.4)	0.72 (0.48, 1.09)	0.75 (0.50, 1.13)	56 (3.2)	0.90 (0.68, 1.19)	0.95 (0.71, 1.25)
≥60 min/week	11.3 (1.8)	83 (11.1)	1.13 (0.90, 1.41)	1.25 (0.99, 1.55)	22 (3.0)	1.07 (0.69, 1.65)	1.19 (0.77, 1.84)	36 (4.8)	1.06 (0.75, 1.49)	1.12 (0.80, 1.58)
p for linear trend			0.12	0.05		0.48	0.32		0.46	0.35
Total cycling										
0 min/week	11.1 (2.1)	1308 (14.0)	1	1	379 (4.1)	1	1	540 (5.8)	1	1
1 – 59 min/week	11.5 (1.8)	236 (8.9)	0.87 (0.76, 1.00)*	0.90 (0.79, 1.04)	72 (2.7)	0.95 (0.74, 1.23)	0.99 (0.76, 1.23)	105 (4.0)	0.86 (0.70, 1.06)	0.90 (0.73, 1.11)
≥60 min/week	11.4 (1.7)	126 (9.2)	1.00 (0.83, 1.21)	1.11 (0.91, 1.32)	34 (2.5)	1.00 (0.70, 1.43)	1.10 (0.77, 1.57)	55 (4.0)	0.92 (0.69, 1.22)	0.98 (0.74, 1.30)
p for linear trend			0.08	0.26		0.36	0.18		0.81	0.51

*p <.05, **p < .01, ***p<.001; 95% CI: 95% confidence interval; FU: follow-up

[a]Adjusted for age, sex, education level and social class

[b]Further adjusted for smoking status, family history of cancer or cardiovascular disease, and all other physical activity

For peer review only - http://bmjopen.bmj.com/site/about/guidelines.xhtml

Table 5: Associations between time spent cycling (total and sub domains; min/week) and physical activity (MET.hr/week) in 13,346 participants

		Leisure Time PA[a] (MET.hr/week)		Total MVPA[b] (MET.hr/week)		Total PA[c] (MET.hr/week)	
	N	Mean (SD)	Regression coefficient (95% CI)[d]	Mean (SD)	Regression coefficient (95% CI)[d]	Mean (SD)	Regression coefficient (95% CI)[d]
Commuter cycling							
0 miles/week (0 min/week)	12484	39.4 (37.7)	0	61.9 (52.3)	0	82.1 (44.6)	0
0.01-9.99 miles/week (1-59 min/week)	587	35.3 (32.4)	-1.6 (-4.7, 1.4)	82.4 (58.0)	12.2 (8.2, 16.2)***	104.9 (46.8)	11.8 (8.3, 15.3)***
≥10 miles/week (≥60 min/week)	275	42.9 (37.6)	1.9 (-2.5, 6.3)	116.6 (63.0)	35.3 (29.5, 41.0)***	128.6 (49.2)	29.7 (24.7, 34.7)***
All utility cycling							
0 miles/week (0 min/week)	10462	38.2 (37.1)	0	60.5 (51.7)	0	81.2 (44.2)	0
0.01-9.99 miles/week (1-59 min/week)	2237	42.9 (38.5)	3.7 (2.1, 5.4)***	69.6 (55.4)	5.5 (3.3, 7.7)***	89.2 (46.9)	4.4 (2.5, 6.3)***
≥10 miles/week (≥60 min/week)	647	46.7 (39.3)	6.9 (3.9, 9.8)***	99.0 (60.4)	25.0 (21.2, 28.9)***	112.1 (50.0)	20.2 (16.9, 23.6)***
Leisure-time cycling							
0 min/week	10843	37.3 (36.5)	0	60.0 (51.7)	0	80.5 (44.1)	0
1-59 min/week	1756	41.0 (35.4)	4.2 (2.3, 6.1)***	73.8 (53.9)	4.7 (2.3, 7.2)***	95.3 (45.3)	4.9 (2.8, 7.0)***
≥60 min/week	747	64.6 (46.6)	25.6 (22.9, 28.2)***	98.0 (61.5)	27.4 (23.9, 31.0)***	110.2 (52.5)	21.9 (18.9, 25.0)***
Total cycling							
0 min/week	9316	37.2 (36.6)	0	58.5 (50.8)	0	79.3 (43.6)	0
1-59 min/week	2654	39.7 (35.8)	3.2 (1.6, 4.9)***	67.0 (53.1)	2.9 (0.8, 5.0)*	88.3 (45.0)	2.6 (0.8, 4.4)*
≥60 min/week	1376	52.4 (43.6)	14.2 (12.2, 16.3)***	94.4 (60.5)	24.1 (21.3, 26.8)***	108.7 (50.7)	19.5 (17.1, 21.8)***

*p <.05, **p < .01, ***p<.001
SD: standard deviation; 95% CI: 95% confidence interval; MVPA: moderate-to-vigorous physical activity; PA: physical activity
[a]Computed as the sum of all moderate-to-vigorous leisure-time physical activity
[b]Computed as the sum of all moderate-to-vigorous physical activity across all domains (leisure-time, household, work, commute)
[c]Computed as the sum of all light and moderate-to-vigorous physical activity across all domains (leisure-time, household, work, commute)
[d]Adjusted for age, sex, education and social class

For peer review only - http://bmjopen.bmj.com/site/about/guidelines.xhtml

Appendix Part 1

Physical Activity Questions at First Health Assessment

In a typical week during the past 12 months, how many hours did you spend on each of the following activities? (Put ‘0’ if none)

Walking, including walking to work and during leisure time
In summer ___ hours per week
In winter ___ hours per week

Cycling, including cycling to work and during leisure time
In summer ___ hours per week
In winter ___ hours per week

Other physical exercise such as keep fit, aerobics, swimming, jogging
In summer ___ hours per week
In winter ___ hours per week

For peer review only

Appendix Part 2

Table A1: Baseline characteristics (first health assessment) of the cohort (n=22,450) by all- cause, cardiovascular and cancer mortality outcomes; values are mean (SD) unless otherwise indicated

Baseline Characteristics	All-cause mortality		Cardiovascular mortality		Cancer mortality	
	Deceased (n=4380)	Survivor (n=18,070)	Deceased (n=1379)	Survivor (n=21071)	Deceased (n=1639)	Survivor (n=20811)
Follow-up years	10.5 (4.6)	16.5 (1.3)***	10.2 (4.7)	15.7 (2.9)***	10.0 (4.4)	15.8 (2.8)***
Male, N (%)	2395 (54.7)	7746 (42.9)***	782 (56.7)	9359 (44.4)***	914 (55.8)	9227 (44.3)***
Age (years)	66.1 (7.7)	56.1 (8.5)***	67.7 (6.7)	57.5 (9.0)***	63.6 (8.2)	57.6 (9.2)***
Education Level, N (%)						
No formal qualification	2049 (46.8)	5957 (33.0)	671 (48.7)	7335 (34.8)	695 (42.4)	7311 (35.1)
GCSE or equivalent	353 (8.1)	2017 (11.2)	105 (7.6)	2265 (10.8)	148 (9.0)	2222 (10.7)
A-Level or equivalent	1617 (36.9	7511 (41.6)	495 (35.9)	8633 (41.0)	654 (39.9)	8474 (40.7)
Degree or equivalent	361 (8.2)	2585 (14.3)***	108 (7.8)	2838 (13.5)***	8.7 (142)	2804 (13.5)***
Social class, N (%)						
Professional	220 (5.0)	1354 (7.5)	77 (5.6)	1497 (7.1)	79 (4.8)	1495 (7.2)
Managerial / technical	2310 (52.7)	9570 (53.0)	719 (52.1)	11161 (53.0)	834 (50.9)	54.1 (11046)
Skilled / partially skilled labour	1642 (37.5)	6568 (36.4)	502 (36.4)	7708 (36.6)	648 (39.5)	7562 (36.3)
Unskilled labour	208 (4.8)	578 (3.2)***	81 (5.9)	705 (3.4)***	78 (4.8)	708 (3.4)***
Family history of CVD, N (%)	2257 (51.5)	2123 (50.2)	756 (54.8)	10570 (50.2)**	797 (48.6)	10529 (50.6)
Family history of cancer, N (%)	1706 (39.0)	7039 (39.0)	901 (65.3)	12799 (60.8)**	709 (43.3)	38.6 (8036)***
Smoking Status, N (%)						
Current	651 (14.9)	1991 (11.0)	205 (14.9)	2437 (11.6)	281 (17.1)	2361 (11.3)
Former	2181 (49.8)	7137 (39.5)	706 (51.2)	8612 (40.9)	802 (48.9)	8516 (40.9)
Never	1548 (35.3)	8942 (49.5)***	468 (33.9)	10022 (47.6)***	556 (33.9)	9934 (47.7)***
Hypertensive Medication (yes)	1278 (29.2)	2396 (13.3)***	517 (37.5)	3157 (15.0)**	360 (22.0)	11279 (78.0)***
Lipid Medication (yes)	58 (1.6)	191 (1.1)	28 (2.0)	221 (1.1)**	15 (0.9)	234 (1.1)
Total energy intake (kj/day)	8736.4 (2527.9)	8611.4 (2513.5)**	8744.4 (2547.8)	8628.5 (2514.6)	8809.5 (2552.9)	8621.9 (2513.4)**
Units of alcohol (units/wk)	7.1 (10.6)	7.2 (9.2)	6.7 (10.0)	7.2 (9.5)*	8.0 (11.5)	7.1 (7.0)

*p < 0.05; **p < 0.005; ***p < 0.001

For peer review only - http://bmjopen.bmj.com/site/about/guidelines.xhtml

Table A2: Descriptive characteristics of participants [N (%)] at second health assessment (n=13346) by cycling (yes, no)

Baseline Characteristics	Commuter Cycling			All Utility Cycling			Leisure-time Cycling		
	None	Some	OR (95% CI)	None	Some	OR (95% CI)	None	Some	OR (95% CI)
Sex									
Male	5395 (93.0)	405 (7.0)	1	4424 (76.3)	1376 (23.7)	1	4555 (78.5)	1245 (21.5)	1
Female	7089 (93.9)	457 (6.1)	0.77 (0.66, 0.89)	6038 (80.19)	1508 (20.0)	0.83 (0.76, 0.91)	5858 (83.3)	1258 (16.7)	0.63 (0.56, 0.70)
Age (yrs)									
40 – 54	3138 (87.3)	456 (12.7)	1	2624 (73.07)	970 (27.0)	1	2534 (70.5)	1060 (29.5)	1
55 – 64	4253 (92.9)	325 (7.1)	0.50 (0.42, 0.58)	3524 (77.0)	1054 (23.0)	0.94 (0.84, 1.04)	3651 (79.8)	927 (20.3)	0.63 (0.25, 0.34)
≥65	5093 (98.4)	81 (1.6)	0.10 (0.08, 0.13)	4314 (83.4)	860 (16.6)	0.90 (0.79, 1.03)	4658 (90.0)	516 (10.0)	0.29 (0.25, 0.34)
Education Level									
Degree or equivalent	1807 (92.7)	143 (7.3)	1	1487 (76.3)	463 (23.7)	1	1493 (76.6)	457 (23.4)	1
A-Level or equivalent	5237 (93.8)	349 (6.3)	0.81 (0.66, 1.00)	4404 (78.8)	1182 (21.2)	0.90 (0.79, 1.03)	4440 (79.5)	1146 (20.5)	0.94 (0.82, 1.07)
GCSE or equivalent	1394 (93.9)	91 (6.1)	0.78 (0.59, 1.00)	1196 (80.5)	289 (19.5)	0.82 (0.69, 0.98)	1196 (80.5)	289 (19.5)	0.86 (0.72, 1.03)
No formal qualification	4046 (93.6)	279 (6.5)	1.01 (0.79, 1.29)	3375 (78.0)	950 (22.0)	1.05 (0.91, 1.22)	3714 (85.9)	611 (14.1)	0.75 (0.64, 0.88)
Social Class									
Professional	932 (93.1)	69 (6.9)	1	764 (76.3)	237 (23.7)	1	775 (77.4)	226 (22.6)	1
Managerial / technical	6990 (95.2)	350 (4.8)	0.72 (0.54, 0.95)	5927 (80.8)	1413 (19.3)	0.78 (0.66, 0.93)	5973 (81.4)	1357 (18.6)	0.85 (0.72, 1.05)
Skilled / partially skilled labour	4147 (91.2)	400 (8.8)	1.36 (1.01, 1.82)	3438 (75.6)	1109 (24.4)	1.03 (0.87, 1.23)	3708 (81.6)	839 (18.5)	0.86 (0.72, 1.04)
Unskilled labour	415 (90.6)	43 (9.4)	1.60 (1.05, 2.45)	333 (72.7)	125 (27.3)	1.25 (0.96, 1.63)	387 (84.5)	71 (15.5)	0.79 (0.58, 1.08)
Paid Employment									
Yes	6306 (88.0)	6178 (100.0)	n/a	5148 (71.8)	2020 (28.2)	1	5118 (76.0)	1617 (24.0)	1
No	862 (12.0)	0 (0)		5314 (86.0)	864 (14.0)	2.33 (2.09, 2.60)	5037 (86.9)	757 (13.1)	1.14 (1.02, 1.28)

For peer review only

Table A3: Associations between demographic characteristics of participants at the first health assessment (n=22,450) and all-cause, cardiovascular disease and cancer mortality

Demographic characteristics		N (%)	All-cause mortality		CVD mortality		Cancer mortality	
			No. deaths	Crude mortality rate (95%CI)	No. deaths	Crude mortality rate (95%CI)	No. deaths	Crude mortality rate (95%CI)
Sex	Male	10,141 (45.2)	2395	1.0	782	1.0	914	1.0
	Female	12,309 (54.8)	1985	0.62 (0.58, 0.66)	597	0.61 (0.55, 0.68)	725	0.63 (0.57, 0.70
Age (years)	40 – 54	9043 (40.3)	462	1.0	90	1.0	270	1.0
	55 – 64	6936 (30.9)	1044	3.29 (2.94, 3.7)	249	3.7 (2.9, 4.7)	537	2.7 (2.3, 3.2)
	≥65	6471 (28.8)	2874	14.8 (13.4, 16.5)	1040	19.0 (15.3, 23.7)	832	4.8 (4.2, 5.5)
Education Level	Degree or equivalent	8006 (35.7)	8006	1.0	671	1.0	695	1.0
	A-level	2370 (10.6)	2370	0.51 (0.45, 0.58)	105	0.51 (0.41, 0.63)	148	0.70 (0.58, 0.84)
	GCSE	9128 (40.7)	9128	0.63 (0.58, 0.67)	495	0.63 (0.56, 0.71)	654	0.81 (0.73, 0.91)
	No formal qualification	2946 (13.1)	2946	0.41 (0.36, 0.46)	108	0.42 (0.34, 0.10)	142	0.53 (0.44, 0.64)
Social Class	Professional	1574 (7.0)	220	1.0	77	1.0	79	1.0
	Managerial / technical	11880 (52.9)	2310	1.49 (1.28, 1.73)	719	1.25 (0.98, 1.59)	834	1.42 (1.13, 1.81)
	Skilled / partially skilled labour	8210 (36.6)	1642	1.54 (1.32, 1.79)	502	1.27 (0.99, 1.62)	648	1.62 (1.28, 2.06)
	Unskilled labour	786 (3.5)	208	2.21 (1.79, 2.74)	81	2.23 (1.61, 3.09)	78	2.08 (1.51, 2.89)
Paid employment	No	10958 (48.8)	3421	1.0	1161	1.0	1125	1.0
	Yes	11492 (51.2)	959	0.20 (0.19, 0.22)	218	0.16 (0.14, 0.19)	514	0.41 (0.37-0.46)

*p <.05, **p < .01, ***p<.001

CVD: Cardiovascular disease; 95% CI: 95% Confidence intervals

For peer review only

STROBE 2007 (v4) Statement—Checklist of items that should be included in reports of *cohort studies*

Section/Topic	Item #	Recommendation	Reported on page #
**Title and abstract**	1	(*a*) Indicate the study's design with a commonly used term in the title or the abstract	1
		(*b*) Provide in the abstract an informative and balanced summary of what was done and what was found	2
**Introduction**			
Background/rationale	2	Explain the scientific background and rationale for the investigation being reported	4
Objectives	3	State specific objectives, including any prespecified hypotheses	4
**Methods**			
Study design	4	Present key elements of study design early in the paper	5
Setting	5	Describe the setting, locations, and relevant dates, including periods of recruitment, exposure, follow-up, and data collection	5
Participants	6	(*a*) Give the eligibility criteria, and the sources and methods of selection of participants. Describe methods of follow-up	5
		(*b*) For matched studies, give matching criteria and number of exposed and unexposed	n/a
Variables	7	Clearly define all outcomes, exposures, predictors, potential confounders, and effect modifiers. Give diagnostic criteria, if applicable	5-6, 13
Data sources/ measurement	8*	For each variable of interest, give sources of data and details of methods of assessment (measurement). Describe comparability of assessment methods if there is more than one group	5-6, 13
Bias	9	Describe any efforts to address potential sources of bias	5-6
Study size	10	Explain how the study size was arrived at	5
Quantitative variables	11	Explain how quantitative variables were handled in the analyses. If applicable, describe which groupings were chosen and why	6-7
Statistical methods	12	(*a*) Describe all statistical methods, including those used to control for confounding	6-7
		(*b*) Describe any methods used to examine subgroups and interactions	n/a
		(*c*) Explain how missing data were addressed	5
		(*d*) If applicable, explain how loss to follow-up was addressed	n/a
		(*e*) Describe any sensitivity analyses	6-7
**Results**			

For peer review only

Participants	13*	(a) Report numbers of individuals at each stage of study—eg numbers potentially eligible, examined for eligibility, confirmed eligible, included in the study, completing follow-up, and analysed	5
		(b) Give reasons for non-participation at each stage	5
		(c) Consider use of a flow diagram	n/a
Descriptive data	14*	(a) Give characteristics of study participants (eg demographic, clinical, social) and information on exposures and potential confounders	5, 7, 14
		(b) Indicate number of participants with missing data for each variable of interest	5
		(c) Summarise follow-up time (eg, average and total amount)	5
Outcome data	15*	Report numbers of outcome events or summary measures over time	6
Main results	16	(*a*) Give unadjusted estimates and, if applicable, confounder-adjusted estimates and their precision (eg, 95% confidence interval). Make clear which confounders were adjusted for and why they were included	15, 16
		(*b*) Report category boundaries when continuous variables were categorized	n/a
		(*c*) If relevant, consider translating estimates of relative risk into absolute risk for a meaningful time period	n/a
Other analyses	17	Report other analyses done—eg analyses of subgroups and interactions, and sensitivity analyses	7
**Discussion**			
Key results	18	Summarise key results with reference to study objectives	8-9
**Limitations**			
Interpretation	20	Give a cautious overall interpretation of results considering objectives, limitations, multiplicity of analyses, results from similar studies, and other relevant evidence	9
Generalisability	21	Discuss the generalisability (external validity) of the study results	9
**Other information**			
Funding	22	Give the source of funding and the role of the funders for the present study and, if applicable, for the original study on which the present article is based	10

*Give information separately for cases and controls in case-control studies and, if applicable, for exposed and unexposed groups in cohort and cross-sectional studies.

Note: An Explanation and Elaboration article discusses each checklist item and gives methodological background and published examples of transparent reporting. The STROBE checklist is best used in conjunction with this article (freely available on the Web sites of PLoS Medicine at http://www.plosmedicine.org/, Annals of Internal Medicine at http://www.annals.org/, and Epidemiology at http://www.epidem.com/). Information on the STROBE Initiative is available at www.strobe-statement.org.

For peer review only
